# Effects of the scope of practice on family physicians: a systematic review

**DOI:** 10.1186/s12875-020-01328-1

**Published:** 2021-01-08

**Authors:** Hervé Tchala Vignon Zomahoun, Isabelle Samson, Jasmine Sawadogo, José Massougbodji, Amédé Gogovor, Ella Diendéré, Frédéric Turgeon, France Légaré

**Affiliations:** 1 VITAM, Research Center in Sustainable Health, CIUSSS de la Capitale-Nationale, Quebec, QC Canada; 2 Health and Social Services Systems, Knowledge Translation and Implementation Component of the Quebec SPOR-SUPPORT Unit, Université Laval, Quebec, QC Canada; 3 Department of Social and Preventive Medicine, Université Laval, Quebec, QC Canada; 4 Centre intégré universitaire de santé et de services sociaux de la Capitale-Nationale (CIUSSS-CN), Quebec, QC Canada; 5 School of Physical & Occupational Therapy, Faculty of medicine and Health Science, McGill University, Montreal, QC Canada; 6 Department of Family Medicine and Emergency Medicine, Université Laval, QC, Canada; 7 Groupe de Médecine Familiale – Quatre-Bourgeois, Quebec, QC Canada; 8 First Nations of Quebec and Labrador Health and Social Services Commission, Québec, Canada; 9 Tier 1 Canada Research Chair in Shared Decision Making and Knowledge Translation, Université Laval, Quebec, QC Canada; 10 Quebec College of Family Physicians, Laval, QC Canada

**Keywords:** Scope of practice, Family physician, Systematic review, Family medicine

## Abstract

**Background:**

Family medicine is a branch of medicine that manages common and long-term illnesses in children and adults. Family physicians in particular play a major role and their scope of practice is expected to impact patient and population. However, little is known about its impact on physicians. We aimed to assess the effects of scope of practice on family physician outcomes.

**Methods:**

We performed a systematic review that we reported using PRISMA guidelines. For the inclusion criteria, any study exploring an association between the scope of practice and physician outcomes was considered. Three bibliographic databases Medline, Embase, and ERIC were consulted through OVID interface from their respective inceptions to November, 2020. Two reviewers independently selected studies, extracted data and assessed the risk of bias of studies using appropriate tools. We conducted data synthesis using a narrative form. GRADE was used for evaluating quality of cumulative evidence.

**Results:**

In total, we included 12 studies with 38,732 participants from 6927 citations identified. Eleven of them were cross-sectional, and one was a cohort study with acceptable methodological quality. We found that: 1) family physicians with diverse clinical and nonclinical activities significantly improve their job satisfaction (*p*<0.05); 2) family physicians with a variety of clinical practices significantly improve their competences and health status (*p*<0.05); 3) family physicians who perform clinical procedures (mainly extended to gynecological procedures) significantly improve their psychosocial outcomes (e.g., job satisfaction) (*p*<0.05); and 4) some associations are not statistically significant (e.g., relation between variety of practice settings and outcomes). We observed that the evidence available has a very low level.

**Conclusions:**

Our findings suggest that the scope of practice may be favorably associated with some family physician outcomes but with a very low level of evidence available. Based on these findings, healthcare system managers could monitor the scope of practice among family physicians and encourage future research in this field.

**Systematic review registration:**

Our protocol was registered under the number CRD42019121990 in PROSPERO.

## Background

Family medicine is a branch of medicine that manages common and long-term illnesses in children and adults, focusing on overall health and well-being [1]. Many healthcare professionals contribute to this field to respond to the varying needs of patients. However, family physicians in particular play a major role in healthcare system. It was strongly showed that people that receive care from primary care physicians improve their health outcomes increasing the life expectancy, and reducing the rate of low birth weight and the total and cause-specific mortality at the state level [2]. Primary care physicians include family physicians, general internists, and general pediatricians in the United States of America; and family physicians or general practitioners in most other developed countries [2]. Family physicians could be more numerous than other medical specialists in the healthcare system. For example, Canadian family physicians with or without a focused practice represented 50.4% of all medical specialists in 2017 [3]. Family physicians must acquire and maintain a wide range of skills in health promotion, leadership, collaboration and communication to better respond to the needs of patients and their communities [4]. The full scope of their nonclinical practice may include teaching/educating, contributing to research as investigators or research subjects, and administering mainly health facility committees and management practices [5]. The scope of their clinical practice may include inpatient care, emergency care and minor clinical procedures, and ambulatory care [6].

Some determinants, such as age, gender, and setting, may explain the scope of practice among family physicians and may help to clarify the realities of practice for family physicians. For example, in a rural setting in the USA, younger physicians reported a broader scope of practice than older physicians did [7]. The scope of practice among women and men physicians in Canada appears similar in the same settings but is broader in rural settings [8]. It was also demonstrated that the large scope of practice offered by family medicine is the main reason why graduating medical students choose to pursue a career in this field [9, 10].

However, we do not know the practices that can impact outcomes for family physician. More specifically, we know little about how different scopes of practice influence family physician outcomes, such as the maintenance or improvement of their clinical skills, practice performance, or health status. This information could guide family physicians in their career decisions as well as decisions about academic training and continuing medical education. It could also inform policy decisions about the broad scope of family physician practice. Thus, we aimed to assess the effects of the scope of practice on family physician outcomes.

## Methods

We conducted a systematic review following the methodology recommended in the Cochrane collaboration handbook [11] and reported it following the Preferred Reporting Items for Systematic Reviews and Meta-Analyses (PRISMA) guidelines [12]. The protocol of our review was registered under the number CRD42019121990 in PROSPERO [13].

### Eligibility cr iteria

We used a PICOS approach (P = population, I = intervention or exposure, C = comparison, O = outcomes, S = study design) to define the eligibility criteria with the collaboration of our content expert (IS). These criteria are described below.

#### Population

Participants had to be family physicians regardless of their characteristics.

#### Intervention or exposure

Any type of scope of practice as defined in our introduction section and used as an exposure in the study identified.

#### Comparison

Lower levels of the scope of practice were considered comparators (e.g., intra- and extra-hospital activities versus intra-hospital activities).

#### Outcomes

Any outcome related to family physicians included, but not limited to, physician performance (e.g., quality of care), clinical competences (e.g., maintenance of skills over time), psychosocial outcomes (e.g., medical knowledge, physician satisfaction), and health status (e.g., physician well-being).

#### Study design

Randomized controlled trials, quasi-experimental trials, cohort studies, case-control studies, and cross-sectional studies were considered.

### Information sources and search strategies

An information specialist performed the literature search in three bibliographic databases, including Medline (Inception date, 1946), Embase (Inception date, 1974), and ERIC (Inception date, 1966) though the OVID interface, from their respective inceptions to November 2020 (see Additional file 1). The search strategy was reviewed by another information specialist using the tool *Peer Review of Electronic Search Strategies* [14] and was discussed with the scientist leading the review and with our content expert. The following main concepts were considered: scope of practice, family physician outcomes, and study designs of interest. Moreover, we consulted the list of references of included studies for additional relevant studies. As we considered only published studies, the gray literature has not been consulted. Animal studies were excluded from the literature search. Language restrictions have not been applied.

#### Study selection process

The process of study selection included four steps. In step 1, the pilot selection of studies was conducted independently by two reviewers on 10% of the total unique references identified. This pilot enabled reviewers to have a shared understanding of eligibility criteria for the main study selection process. In step 2, following a conclusive pilot, each reviewer performed an independent selection by title/abstract. In step 3, after a consensus was reached, all references retained were considered for selection by full text. Corresponding or first authors of studies were contacted by email to obtain missing information or clarification when needed. The two reviewers discussed and resolved any disagreement or else refereed by a third reviewer at steps 2 and 3. In step 4, the included studies were discussed by the reviewers and our content expert for the final selection.

#### Data collection process

We developed a data extraction form on which the following variables were considered: *study characteristics*, including name of the first author, the study design, the year of publication, and the country where the study was conducted; *population characteristics*, including the total number of family physicians, sex, mean age, the number of years of clinical experience, and practice settings; *characteristics of scope of practice*, including the number of family physician practices considered in studies, the names and definitions of these practices, the description of services offered by family physicians, and the measurement and category of the scope of practice; and *outcomes characteristics*, including the name and category of outcomes based on the taxonomy of Cochrane Effective Practice and Organization of Care (EPOC) [15], its scale, the type of association measures (e.g., odds ratio, mean difference), the crude and adjusted association measures, the amplitude of the measure of association and its 95% confidence interval. After a conclusive pilot phase, two reviewers independently performed data extraction using our data extraction form. Any disagreement about the extracted data was discussed by the two reviewers.

#### Risk of bias in individual studies

The risk of bias in the studies was independently assessed by two reviewers using appropriate tools according to the study design. Since only cohort and cross-sectional studies were identified, we used the Newcastle-Ottawa Scale for cohort studies [16], and the Joanna Briggs Institute tool for cross-sectional studies [17]. Disagreements were discussed by the two reviewers and refereed by a third author when needed.

### Data synthesis

We described the process of study selection using frequency counts. The extracted data were synthesized in narrative form with respect to the studies, populations, exposure, and outcome characteristics. The effects of the different types of scope of practice on family physician outcomes were also narratively synthesized using the association measures with their 95% confidence intervals reported. In the case these latter were missing, we calculated them when sufficient data were available [18]. The level of risk of bias was taken into account in the interpretation of the observed effects. Meta-analysis was not performed because we anticipated high heterogeneity between studies. Moreover, publication bias and subgroup analyses were not conducted.

### Cumulative evidence quality

For each outcome studied, the quality of cumulative evidence with the Grading of Recommendations Assessment, Development and Evaluation (GRADE) [19] was evaluated by one author with experience. All GRADE criteria were used for this evaluation. Considering study designs, observational studies or non-randomized trials were rated (score = + 2) and randomized trials (score = + 4) [19]. This score was downgraded or unchanged depending of the rating of the risk of bias, inconsistency, indirectness, imprecision, or publication bias [19]. Each criterion was rated none (score = 0), borderline (score = − 0.5), serious (score = − 1), and very serious (score = − 2). The score obtained from the study design was upgraded (+ 1) when the association measure was strong or showed a dose-response in the absence of plausible residual confounding bias [19]. The overall certainty was rated very low, low, moderate or high using the final score [19].

## Results

### Description of studies selection process

**Fig. 1:**
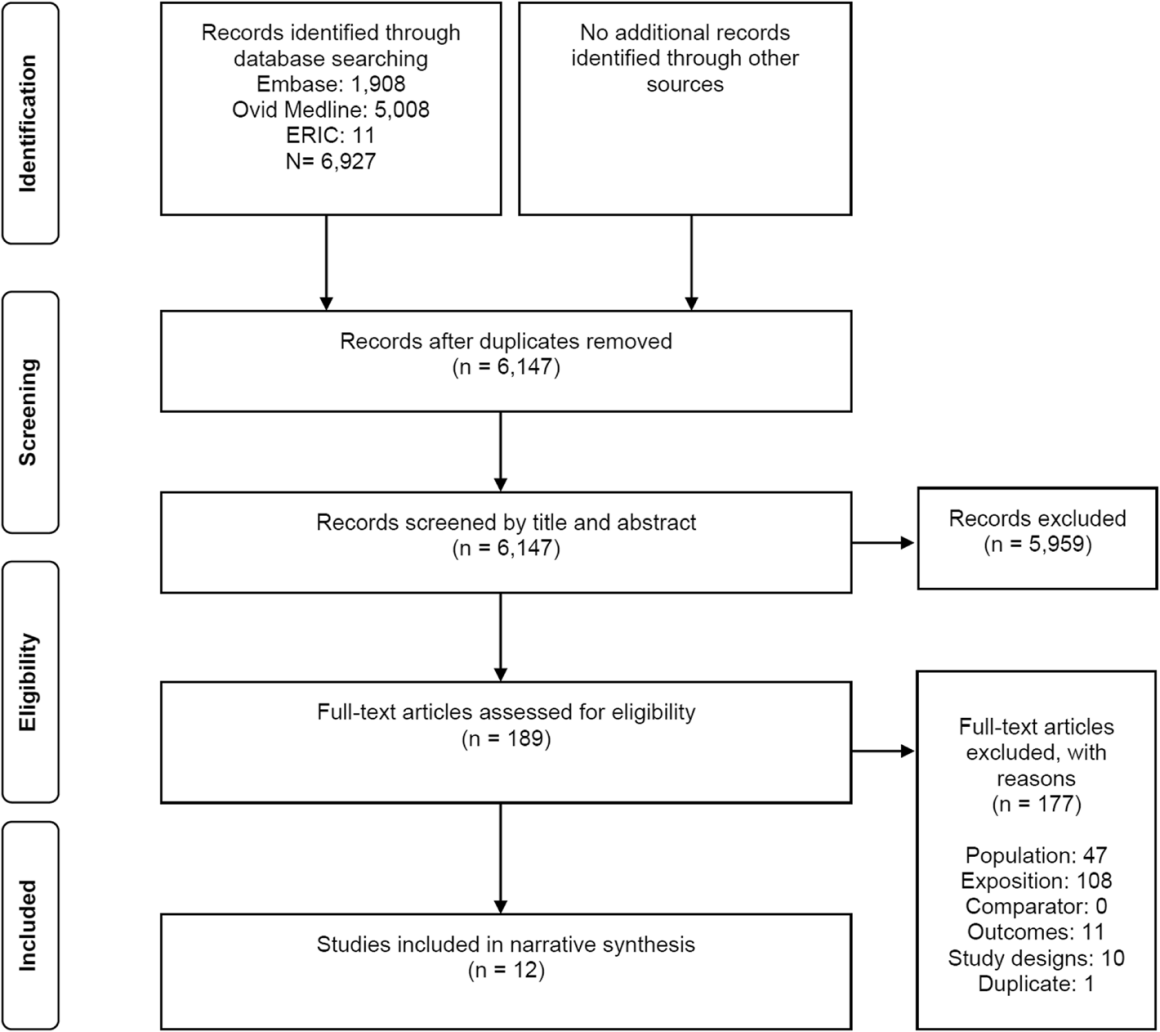
PRISMA diagram for the studies selection process

In total, we included 12 studies with 38,732 participants from 6927 citations identified from literature search strategies (see Fig. 1) [20–31]. Among these included studies, eleven were cross-sectional studies [20–24, 26–31], and one was a cohort study [25]. The included studies were published between 1996 [27] and 2020 [30, 31] and were conducted in the USA (*N* = 6) [21–23, 27, 28, 31], in Canada (*N* = 4) [20, 24, 26, 29], in Portugal (*N* = 1) [25], and in Switzerland (*N* = 1) [30].

### Characteristics of the study population

The majority of study participants were family physicians with experience except in one study on new family physicians [28]. The mean age of the participants was reported in seven studies and varied from 35.9 [28] to 55.0 [22] (median = 53.1). Gender information was reported in 11 studies [20–26, 28–31] with the percentage of women varying from 0.11 [29] to 0.59 [28] (median = 0.44). The clinical experience in years was rarely reported in the included studies, except for three studies in which this information was reported differently: a mean age of 16.3 [20], a median age of 22.0, [20] and a percentage, i.e., a total of 41.0% of participants had at least 21 years of experience in practice [20].

### Characteristics of the scope of practice

Table 1 shows a description of the scope of practice as reported in the included studies. The definition of the scope of practice was explicitly reported in six studies [22–25, 28, 31], and covered the dimensions of scope, such as clinical procedures [22–24, 28], clinical practices [25, 28, 31], clinical and nonclinical activities [24, 30], and practice settings [25, 28]. It was self-reported in nine studies [20, 21, 23–26, 28, 30, 31], or reported with objective measures (e.g., direct observation or administrative database) in two studies [22, 27], with a mixed approach (subjective and objective measures) used in one study [29].

**Table 1:** Data extraction of studies included

Study 1. Authors 2. Publication year 3. Study design 4. Country	Population characteristics		Exposure and outcome characteristics		Effect of exposure on outcome
	1. Initial sample size 2. Profile 3. Clinical experience	4. Age (in years) 5. Number of women 6. Settings	1. Exposure name 2. Exposure measurement 3. Exposure category	4. Outcome name 5. Outcome measurement 6. Outcome category	1. Effect estimation methods 2. Crude effect (95%CI; *p*-value) 3. Adjusted effect (95%CI; *p*-value) 4. Sample size analyzed
1. Ward 2. 2020 3. Cross-sectional 4. USA	1. *N* = 2740 2. Family physicians 3. NR	4. Mean age = NR (NR) 5. *n* = 1228 6. American Board of Family Medicine	1. Scope of practice 2. Scope of Practice for Primary Care (SP4PC) Score 3. Clinical practice	4. Burnout 5. Self-reported two validated single-item questions 6. Physician clinical status	1. Logistic regression models 2. NR (NR; NR) 3. OR = 0.98 (0.96–1.01; NR) 4. *N* = 2740
1. Mooser 2. 2020 3. Cross-sectional 4. Switzerland	1. *N* = 199 2. Family physicians 3. NR	4. Mean age = 55.0 (8.0) 5. *n* = 44 6. Primary care physicians’ associations	1. Administrative overload 2. 60-question postal questionnaire 3. Non-clinical activities	4. Loss of meaning in work 5. 60-question postal questionnaire 6. Psychosocial outcomes	1. Logistic regression models 2. OR = 4.53 (2.28–9.01; NR) 3. OR = 4.18 (2.04–8.58; NR) 4. *N* = 190
			1. Teaching activity 2. 60-question postal questionnaire 3. Non-clinical activities	4. Loss of meaning in work 5. 60-question postal questionnaire 6. Psychosocial outcomes	1. Logistic regression models 2. OR = 0.52 (0.29; 0.91; NR) 3. OR = 0.50 (0.27–0.90; NR) 4. *N* = 190
1. Weidner 2. 2018 3. Cross-sectional 4. USA	1. *N* = 1617 2. Family physicians 3. NR	4. Mean age = 35.9 (4.4) 5. *n* = 948 6. Multiple clinical settings	1. Practicing inpatient medicine 2. Survey items 3. Clinical practice	4. Burnout 5. Maslach Burnout Inventory 6. Physician clinical status	1. Logistic regression models 2. NR (NR; NR) 3. OR = 0.70 (0.56–0.87; 0.0017) 4. *N* = 1617
			1. Practicing obstetrics 2. Survey items 3. Clinical practice	4. Burnout 5. Maslach Burnout Inventory 6. Physician clinical status	1. Logistic regression models 2. NR (NR; NR) 3. OR = 0.64 (0.47–0.88; 0.0058) 4. *N* = 1617
			1. Pediatric ambulatory care 2. Survey items 3. Clinical practice	4. Burnout 5. Maslach Burnout Inventory 6. Physician clinical status	1. Logistic regression models 2. NR (NR; NR) 3. OR = 0.88 (0.66–1.19; 0.4200) 4. *N* = 1617
1. Rodrigues 2. 2016 3. Cohort 4. Portugal	1. *N* = 421 2. Family physicians 3. NR	4. Median age = 55 5. *n* = 214 6. Primary care and hospital	1. Public and private practice 2. Questionnaire 3. Practice settings	4. Quality of antibiotic prescribing 5. European Surveillance of Antibiotic Consumption 6. Physician performance	1. Generalized linear mixed models 2. NR (NR; NR) 3. OR = 1.13 (0.58–2.22; NR) 4. *N* = 95
			1. Hospital and primary care settings 2. Questionnaire 3. Practice settings	4. Quality of antibiotic prescribing 5. European Surveillance of Antibiotic Consumption 6. Physician performance	1. Generalized linear mixed models 2. NR (NR; NR) 3. OR = 0.76 (0.39–1.49; NR) 4. *N* = 96
			1. Emergency activity 2. Questionnaire 3. Clinical practice	4. Quality of antibiotic prescribing 5. European Surveillance of Antibiotic Consumption 6. Physician performance	1. Generalized linear mixed models 2. NR (NR; NR) 3. OR = 0.29 (0.16–0.54; NR) 4. *N* = 280
1. Nisen 2. 2016 3. Cross-sectional 4. USA	1. *N* = 2329 2. Family physicians 3. ≥21 years, *n* = 961 [11–20], *n* = 779 ≤10 years, *n* = 589	4. Mean age = 55.0 (NR) 5. *n* = 857 6. NR	1. Performs endometrial biopsies 2. Questionnaire 3. Clinical procedures	4. Providing IUD insertion 5. Do you regularly perform IUD insertion? Yes, or no 6. Physician performance	1. Logistic regression models 2. NR (NR; NR) 3. OR = 16.88 (12.21–23.35; NR) 4. NR
			1. Performs endometrial biopsies 2. Questionnaire 3. Clinical procedures	4. Providing Long-acting contraception insertion or removal 5. Do you regularly perform implantable long-acting contraception insertion or removal? Yes, or no 6. Physician performance	1. Logistic regression models 2. NR (NR; NR) 3. OR = 3.90 (2.54–5.95; NR) 4. NR
			1. Performs implant insertions and removals 2. Questionnaire 3. Clinical procedures	4. Providing IUD insertion 5. Do you regularly perform IUD insertion? Yes, or no 6. Physician performance	1. Logistic regression models 2. NR (NR; NR) 3. OR = 9.04 (5.94–13.75; NR) 4. NR
			1. Performs IUD insertion 2. Questionnaire 3. Clinical procedures	4. Providing Long-acting contraception insertion or removal 5. Do you regularly perform implantable long-acting contraception insertion or removal? Yes, or no 6. Physician performance	1. Logistic regression models 2. NR (NR; NR) 3. OR = 8.92 (5.93–13.43; NR) 4. NR
			1. Performs skin procedures 2. Questionnaire 3. Clinical procedures	4. Providing IUD insertion 5. Do you regularly perform IUD insertion? Yes, or no 6. Physician performance	1. Logistic regression models 2. NR (NR; NR) 3. OR = 1.63 (0.96–2.77; NR) 4. NR
			1. Performs skin procedures 2. Questionnaire 3. Clinical procedures	4. Providing Long-acting contraception insertion or removal 5. Do you regularly perform implantable long-acting contraception insertion or removal? Yes, or no 6. Physician performance	1. Logistic regression models 2. NR (NR; NR) 3. OR = 3.14 (1.50–6.59; NR) 4. NR
			1. Provides prenatal care and deliveries 2. Questionnaire 3. Clinical procedures	4. Providing IUD insertion 5. Do you regularly perform IUD insertion? Yes, or no 6. Physician performance	1. Logistic regression models 2. NR (NR; NR) 3. OR = 3.37 (1.99–5.69; NR) 4. NR
			1. Provides prenatal care and deliveries 2. Questionnaire 3. Clinical procedures	4. Providing Long-acting contraception insertion or removal 5. Do you regularly perform implantable long-acting contraception insertion or removal? Yes, or no 6. Physician performance	1. Logistic regression models 2. NR (NR; NR) 3. OR = 1.76 (1.14–2.72; NR) 4. NR
			1. Provides prenatal care no deliveries 2. Questionnaire 3. Clinical procedures	4. Providing IUD insertion 5. Do you regularly perform IUD insertion? Yes, or no 6. Physician performance	1. Logistic regression models 2. NR (NR; NR) 3. OR = 3.40 (1.90–6.10; NR) 4. NR
			1. Provides prenatal care no deliveries 2. Questionnaire 3. Clinical procedures	4. Providing Long-acting contraception insertion or removal 5. Do you regularly perform implantable long-acting contraception insertion or removal? Yes, or no 6. Physician performance	1. Logistic regression models 2. NR (NR; NR) 3. OR = 1.35 (0.75–2.43; NR) 4. NR
1. Peterson 2. 2015 3. Cross-sectional 4. USA	1. *N* = 8838 2. Family physicians 3. NR	4. Mean age = 51.0 (8.5) 5. *n* = 3403 6. Urban settings	1. Clinical activities 2. Scope of Practice for Primary Care (SP4PC) scale 3. Clinical practices	4. Maintenance of family physician certification 5. Maintenance of certification for family physicians’ examination score 6. Physician performance	1. Logistic regression models 2. NR (NR; NR) 3. OR = 1.08 (1.06–1.11; NR) 4. NR
	1. *N* = 2140 2. Family physicians 3. NR	4. Mean age = 51.9 (8.6) 5. *n* = 623 6. Rural settings	1. Clinical activities 2. Scope of Practice for Primary Care (SP4PC) scale 3. Clinical practices	4. Maintenance of family physician certification 5. Maintenance of certification for family physicians’ examination score 6. Physician performance	1. Logistic regression models 2. NR (NR; NR) 3. OR = 1.11 (1.07–1.16; NR) 4. NR
1. Wenghofer 2. 2009 3. Cross-sectional 4. USA	1. *N* = 532 2. Family physicians 3. NR	4. Mean age = 51 (9.91) 5. *n* = 59 6. clinics and hospitals	1. Focused practice scope 2. Extracted from the College of Physicians and Surgeons of Ontario registry or self-reported by family physicians 3. Clinical practices	4. Managing patients with chronic conditions 5. Multiple-item measure scores on physician ranging from 1 to 4 6. Physician performance	1. Linear regression models 2. NR (NR; NR) 3. Regression coefficient = NR (NR; p>0.05) 4. NR
			1. Focused practice scope 2. Extracted from the College of Physicians and Surgeons of Ontario registry or self-reported by family physicians 3. Clinical practices	4. Providing patients with continuity of care and referrals 5. Multiple-item measure scores on physician ranging from 1 to 4 6. Physician performance	1. Linear regression models 2. NR (NR; NR) 3. Regression coefficient = NR (NR; p>0.05) 4. NR
			1. Focused practice scope 2. Extracted from the College of Physicians and Surgeons of Ontario registry or self-reported by family physicians 3. Clinical practices	4. Providing patients with well care and health maintenance 5. Multiple-item measure scores on physician ranging from 1 to 4 6. Physician performance	1. Linear regression models 2. NR (NR; NR) 3. Regression coefficient = NR (NR; p>0.05) 4. NR
			1. Focused practice scope 2. Extracted from the College of Physicians and Surgeons of Ontario registry or self-reported by family physicians 3. Clinical practices	4. Managing patient records 5. Multiple-item measure scores on physician ranging from 1 to 4 6. Physician performance	1. Linear regression models 2. NR (NR; NR) 3. Regression coefficient = NR (NR; p>0.05) 4. NR
			1. Focused practice scope 2. Extracted from the College of Physicians and Surgeons of Ontario registry or self-reported by family physicians 3. Clinical practices	4. Managing patients with acute conditions and new presentations 5. Multiple-item measure scores on physician ranging from 1 to 4 6. Physician performance	1. Linear regression models 2. NR (NR; NR) 3. Regression coefficient = NR (NR; p>0.05) 4. NR
			1. Holds Active Hospital appointment 2. Extracted from the College of Physicians and Surgeons of Ontario registry or self-reported by family physicians 3. Clinical practices	4. Managing patients with chronic conditions 5. Multiple-item measure scores on physician ranging from 1 to 4 6. Physician performance	1. Linear regression models 2. NR (NR; NR) 3. Regression coefficient = NR (NR; p>0.05) 4. NR
			1. Holds Active Hospital appointment 2. Extracted from the College of Physicians and Surgeons of Ontario registry or self-reported by family physicians 3. Clinical practices	4. Providing patients with continuity of care and referrals 5. Multiple-item measure scores on physician ranging from 1 to 4 6. Physician performance	1. Linear regression models 2. NR (NR; NR) 3. Regression coefficient = NR (NR; p>0.05) 4. NR
			1. Holds Active Hospital appointment 2. Extracted from the College of Physicians and Surgeons of Ontario registry or self-reported by family physicians 3. Clinical practices	4. Providing patients with well care and health maintenance 5. Multiple-item measure scores on physician ranging from 1 to 4 6. Physician performance	1. Linear regression models 2. NR (NR; NR) 3. Regression coefficient = NR (NR; p>0.05) 4. NR
			1. Holds Active Hospital appointment 2. Extracted from the College of Physicians and Surgeons of Ontario registry or self-reported by family physicians 3. Clinical practices	4. Managing patient records 5. Multiple-item measure scores on physician ranging from 1 to 4 6. Physician performance	1. Linear regression models 2. NR (NR; NR) 3. Regression coefficient = 0.08 (0.01–0.15; NR) 4. NR
			1. Holds Active Hospital appointment 2. Extracted from the College of Physicians and Surgeons of Ontario registry or self-reported by family physicians 3. Clinical practices	4. Managing patients with acute conditions and new presentations 5. Multiple-item measure scores on physician ranging from 1 to 4 6. Physician performance	1. Linear regression models 2. NR (NR; NR) 3. Regression coefficient = NR (NR; p>0.05) 4. NR
			1. Episodic care practice/walk-in clinic 2. Extracted from the College of Physicians and Surgeons of Ontario registry or self-reported by family physicians 3. Clinical practices	4. Managing patients with chronic conditions 5. Multiple-item measure scores on physician ranging from 1 to 4 6. Physician performance	1. Linear regression models 2. NR (NR; NR) 3. Regression coefficient = −0.166 (−0.31 - -0.03; NR) 4. NR
			1. Episodic care practice/walk-in clinic 2. Extracted from the College of Physicians and Surgeons of Ontario registry or self-reported by family physicians 3. Clinical practices	4. Providing patients with continuity of care and referrals 5. Multiple-item measure scores on physician ranging from 1 to 4 6. Physician performance	1. Linear regression models 2. NR (NR; NR) 3. Regression coefficient = NR (NR; p>0.05) 4. NR
			1. Episodic care practice/walk-in clinic 2. Extracted from the College of Physicians and Surgeons of Ontario registry or self-reported by family physicians 3. Clinical practices	4. Providing patients with well care and health maintenance 5. Multiple-item measure scores on physician ranging from 1 to 4 6. Physician performance	1. Linear regression models 2. NR (NR; NR) 3. Regression coefficient = NR (NR; p>0.05) 4. NR
			1. Episodic care practice/walk-in clinic 2. Extracted from the College of Physicians and Surgeons of Ontario registry or self-reported by family physicians 3. Clinical practices	4. Managing patient records 5. Multiple-item measure scores on physician ranging from 1 to 4 6. Physician performance	1. Linear regression models 2. NR (NR; NR) 3. Regression coefficient = NR (NR; p>0.05) 4. NR
			1. Episodic care practice/walk-in clinic 2. Extracted from the College of Physicians and Surgeons of Ontario registry or self-reported by family physicians 3. Clinical practices	4. Managing patients with acute conditions and new presentations 5. Multiple-item measure scores on physician ranging from 1 to 4 6. Physician performance	1. Linear regression models 2. NR (NR; NR) 3. Regression coefficient = NR (NR; p>0.05) 4. NR
1. Thind 2. 2009 3. Cross-sectional 4. Canada	1. *N* = 719 2. Family physicians 3. NR	4. Mean age = 48.4 (NR) 5. *n* = 415 6. NR	1. Teaching activities 2. Questionnaire 3. Variety of activities	4. Family physician satisfaction 5. How satisfied are you with your current practice? Score ranged from 1 to 5; Score = 5 very satisfied <5 not very satisfied 6. Psychosocial outcomes	1. Generalized linear mixed models 2. NR (NR; NR) 3. OR = 2.59 (NR; 0.000) 4. *N* = 620
1. Rivet 2. 2007 3. Cross-sectional 4. Canada	1. *N* = 20,507 2. Family physicians 3. NR	4. Mean age = NR (NR) 5. *n* = 7134 6. All except free-standing walk-in clinics, nursing homes, hospital inpatient units, or emergency departments	1. Variety of procedures done 2. List of 18 procedures 3. Clinical procedures	4. Family physician satisfaction 5. Three items of satisfaction in the survey. Score ranged from 3 to 21 6. Psychosocial outcomes	1. Multiple linear regression models 2. NR (NR; NR) 3. Standardized regression coefficient = 0.04 (NR; 0.001) 4. *N* = 16,877
		4. Mean age = NR (NR) 5. *n* = 7134 6. Private offices or clinics, community clinics or health centres, or academic family medicine teaching units	1. Teaching 2. Self-report questionnaire 3. Variety of activities	4. Family physician satisfaction 5. Three items of satisfaction in the survey. Score ranged from 3 to 21 6. Psychosocial outcomes	1. Multiple linear regression models 2. NR (NR; NR) 3. Standardized regression coefficient = 0.52 (NR; 0.000) 4. *N* = 16,877
1. Cavanagh 2. 2006 3. Cross-sectional 4. Canada	1. *N* = 182 2. Family physicians 3. Mean = 16.5 years	4. Mean age = NR (NR) 5. *n* = 79 6. Province of Newfoundland: Urban, Semi urban, and Rural	1. Perform deliveries 2. Adaptation of the questionnaire used by Carroll et al. 3. Clinical procedures	4. Offering maternal serum screening to all pregnant patients 5. Adaptation of the questionnaire used by Carroll et al. 6. Physician performance	1. Chi-square test 2. OR = 4.31 (1.81–10.22; NR) 3. NR (NR; NR) 4. *N* = 119
			1. Perform deliveries 2. Adaptation of the questionnaire used by Carroll et al. 3. Clinical procedures	4. Offering maternal serum screening to all pregnant patients 5. Adaptation of the questionnaire used by Carroll et al. 6. Physician performance	1. Chi-square test 2. OR = 4.87 (1.81–10.22; NR) 3. NR (NR; NR) 4. *N* = 87
1. Eliason 2. 2000 3. Cross-sectional 4. USA	1. *N* = 712 2. Family physicians 3. Median = 22 years	4. Mean age = NR (NR) 5. *n* = 121 6. Multiple settings	1. Inpatient care practices 2. The Schwartz values questionnaire 3. Clinical practices	4. Security (Family physician personal value) 5. The Schwartz values questionnaire 6. Psychosocial outcomes	1. Analysis of variance and regression analysis 2. NR (NR; NR) 3. NR (NR; > 0.05) 4. *N* = 700
			1. Inpatient care practices 2. The Schwartz values questionnaire 3. Clinical practices	4. Hedonism (Family physician personal value) 5. The Schwartz values questionnaire 6. Psychosocial outcomes	1. Analyse of variance and regression analysis 2. NR (NR; NR) 3. NR (NR; > 0.05) 4. *N* = 704
			1. Inpatient care practices 2. The Schwartz values questionnaire 3. Clinical practices	4. Universalism (Family physician personal value) 5. The Schwartz values questionnaire 6. Psychosocial outcomes	1. Analyse of variance and regression analysis 2. NR (NR; NR) 3. NR (NR; > 0.05) 4. *N* = 701
			1. Inpatient care practices 2. The Schwartz values questionnaire 3. Clinical practices	4. Conformity (Family physician personal value) 5. The Schwartz values questionnaire 6. Psychosocial outcomes	1. Analyse of variance and regression analysis 2. NR (NR; NR) 3. NR (NR; > 0.05) 4. *N* = 704
			1. Inpatient care practices 2. The Schwartz values questionnaire 3. Clinical practices	4. Power (Family physician personal value) 5. The Schwartz values questionnaire 6. Psychosocial outcomes	1. Analyse of variance and regression analysis 2. NR (NR; NR) 3. NR (NR; = 0.01) 4. *N* = 703
			1. Inpatient care practices 2. The Schwartz values questionnaire 3. Clinical practices	4. Benevolence (Family physician personal value) 5. The Schwartz values questionnaire 6. Psychosocial outcomes	1. Analyse of variance and regression analysis 2. NR (NR; NR) 3. NR (NR; > 0.05) 4. *N* = 706
			1. Inpatient care practices 2. The Schwartz values questionnaire 3. Clinical practices	4. Self-direction (Family physician personal value) 5. The Schwartz values questionnaire 6. Psychosocial outcomes	1. Analyse of variance and regression analysis 2. NR (NR; NR) 3. NR (NR; > 0.05) 4. *N* = 698
			1. Inpatient care practices 2. The Schwartz values questionnaire 3. Clinical practices	4. Stimulation (Family physician personal value) 5. The Schwartz values questionnaire 6. Psychosocial outcomes	1. Analyse of variance and regression analysis 2. NR (NR; NR) 3. NR (NR; > 0.05) 4. *N* = 699
			1. Inpatient care practices 2. The Schwartz values questionnaire 3. Clinical practices	4. Achievement (Family physician personal value) 5. The Schwartz values questionnaire 6. Psychosocial outcomes	1. Analyse of variance and regression analysis 2. NR (NR; NR) 3. NR (NR; > 0.05) 4. *N* = 703
			1. Inpatient care practices 2. The Schwartz values questionnaire 3. Clinical practices	4. Tradition (Family physician personal value) 5. The Schwartz values questionnaire 6. Psychosocial outcomes	1. Analyse of variance and regression analysis 2. NR (NR; NR) 3. NR (NR; > 0.05) 4. *N* = 695
			1. Multiple practice 2. The Schwartz values questionnaire 3. Clinical practices	4. Satisfaction 5. The Schwartz values questionnaire 6. Psychosocial outcomes	1. Analyse of variance and regression analysis 2. NR (NR; NR) 3. NR (NR; > 0.05) 4. *N* = 712
			1. Teaching medical trainees 2. The Schwartz values questionnaire 3. Diversity of activities	4. Hedonism 5. The Schwartz values questionnaire 6. Psychosocial outcomes	1. Analyse of variance and regression analysis 2. NR (NR; NR) 3. NR (NR; = 0.006) 4. *N* = 704
			1. Teaching medical trainees 2. The Schwartz values questionnaire 3. Diversity of activities	4. Universalism 5. The Schwartz values questionnaire 6. Psychosocial outcomes	1. Analyse of variance and regression analysis 2. NR (NR; NR) 3. NR (NR; > 0.05) 4. *N* = 701
			1. Teaching medical trainees 2. The Schwartz values questionnaire 3. Diversity of activities	4. Conformity 5. The Schwartz values questionnaire 6. Psychosocial outcomes	1. Analyse of variance and regression analysis 2. NR (NR; NR) 3. NR (NR; > 0.05) 4. *N* = 704
			1. Teaching medical trainees 2. The Schwartz values questionnaire 3. Diversity of activities	4. Power 5. The Schwartz values questionnaire 6. Psychosocial outcomes	1. Analyse of variance and regression analysis 2. NR (NR; NR) 3. NR (NR; > 0.05) 4. *N* = 703
			1. Teaching medical trainees 2. The Schwartz values questionnaire 3. Diversity of activities	4. Benevolence 5. The Schwartz values questionnaire 6. Psychosocial outcomes	1. Analyse of variance and regression analysis 2. NR (NR; NR) 3. NR (NR; > 0.05) 4. *N* = 706
			1. Teaching medical trainees 2. The Schwartz values questionnaire 3. Diversity of activities	4. Self-direction 5. The Schwartz values questionnaire 6. Psychosocial outcomes	1. Analyse of variance and regression analysis 2. NR (NR; NR) 3. NR (NR; > 0.05) 4. *N* = 698
			1. Teaching medical trainees 2. The Schwartz values questionnaire 3. Diversity of activities	4. Stimulation 5. The Schwartz values questionnaire 6. Psychosocial outcomes	1. Analyse of variance and regression analysis 2. NR (NR; NR) 3. NR (NR; > 0.05) 4. *N* = 699
			1. Teaching medical trainees 2. The Schwartz values questionnaire 3. Diversity of activities	4. Achievement 5. The Schwartz values questionnaire 6. Psychosocial outcomes	1. Analyse of variance and regression analysis 2. NR (NR; NR) 3. NR (NR; > 0.05) 4. *N* = 703
			1. Teaching medical trainees 2. The Schwartz values questionnaire 3. Diversity of activities	4. Tradition 5. The Schwartz values questionnaire 6. Psychosocial outcomes	1. Analyse of variance and regression analysis 2. NR (NR; NR) 3. NR (NR; > 0.05) 4. *N* = 695
			1. Teaching medical trainees 2. The Schwartz values questionnaire 3. Diversity of activities	4. Security 5. The Schwartz values questionnaire 6. Psychosocial outcomes	1. Analyse of variance and regression analysis 2. NR (NR; NR) 3. NR (NR; = 0.004) 4. *N* = 700
			1. Obstetric practice 2. The Schwartz values questionnaire 3. Clinical practices	4. Hedonism 5. The Schwartz values questionnaire 6. Psychosocial outcomes	1. Analyse of variance and regression analysis 2. NR (NR; NR) 3. NR (NR; = 0.02) 4. *N* = 704
			1. Obstetric practice 2. The Schwartz values questionnaire 3. Clinical practices	4. Universalism 5. The Schwartz values questionnaire 6. Psychosocial outcomes	1. Analyse of variance and regression analysis 2. NR (NR; NR) 3. NR (NR; = 0.02) 4. *N* = 701
			1. Obstetric practice 2. The Schwartz values questionnaire 3. Clinical practices	4. Conformity 5. The Schwartz values questionnaire 6. Psychosocial outcomes	1. Analyse of variance and regression analysis 2. NR (NR; NR) 3. NR (NR; = 0.05) 4. *N* = 704
			1. Obstetric practice 2. The Schwartz values questionnaire 3. Clinical practices	4. Power 5. The Schwartz values questionnaire 6. Psychosocial outcomes	1. Analyse of variance and regression analysis 2. NR (NR; NR) 3. NR (NR; > 0.05) 4. *N* = 703
			1. Obstetric practice 2. The Schwartz values questionnaire 3. Clinical practices	4. Benevolence 5. The Schwartz values questionnaire 6. Psychosocial outcomes	1. Analyse of variance and regression analysis 2. NR (NR; NR) 3. NR (NR; > 0.05) 4. *N* = 706
			1. Obstetric practice 2. The Schwartz values questionnaire 3. Clinical practices	4. Self-direction 5. The Schwartz values questionnaire 6. Psychosocial outcomes	1. Analyse of variance and regression analysis 2. NR (NR; NR) 3. NR (NR; > 0.05) 4. *N* = 698
			1. Obstetric practice 2. The Schwartz values questionnaire 3. Clinical practices	4. Stimulation 5. The Schwartz values questionnaire 6. Psychosocial outcomes	1. Analyse of variance and regression analysis 2. NR (NR; NR) 3. NR (NR; > 0.05) 4. *N* = 699
			1. Obstetric practice 2. The Schwartz values questionnaire 3. Clinical practices	4. Achievement 5. The Schwartz values questionnaire 6. Psychosocial outcomes	1. Analyse of variance and regression analysis 2. NR (NR; NR) 3. NR (NR; > 0.05) 4. *N* = 703
			1. Obstetric practice 2. The Schwartz values questionnaire 3. Clinical practices	4. Tradition 5. The Schwartz values questionnaire 6. Psychosocial outcomes	1. Analyse of variance and regression analysis 2. NR (NR; NR) 3. NR (NR; > 0.05) 4. *N* = 695
			1. Obstetric practice 2. The Schwartz values questionnaire 3. Clinical practices	4. Security 5. The Schwartz values questionnaire 6. Psychosocial outcomes	1. Analyse of variance and regression analysis 2. NR (NR; NR) 3. NR (NR; = 0.004) 4. *N* = 700
1. Vinson 2. 1996 3. Cross-sectional 4. USA	1. *N* = 22 2. Family physicians 3. NR	4. NR (NR) 5. NR 6. Clinics and hospitals	1. Teaching 2. Questionnaire 3. Clinical practices	4. Time spent at work 5. Objective measure 6. Physician performance	1. Paired t test 2. Mean difference = 52 (16–88; = 0.007) 3. NR (NR; NR) 4. *N* = 22
			1. Teaching 2. Questionnaire 3. Clinical practices	4. Number of patients seen per hour 5. Objective measure 6. Physician performance	1. Paired t test 2. Mean difference = −0.6 (−1.1, −0.1; = 0.03) 3. NR (NR; NR) 4. *N* = 22

After the data analysis, we classified in a posteriori the scope of practice into four categories, including the variety of clinical practices, the diversity of clinical procedures, the diversity of family physician activities (clinical and nonclinical), and the variety of practice settings. Therefore, we found that the scope of practice was characterized by the variety of clinical practices in four studies [23, 28, 29, 31], the diversity of clinical procedures performed in three studies [20–22], the diversity of family physician activities (e.g., primary care practices plus clinical teaching) in four studies [24, 26, 27, 30], and the variety of practice settings in which family physicians worked (e.g., hospital plus primary care center versus primary care center) in one study [25].

### Characteristics of family physician outcomes

Many family physician outcomes were identified and measured with self-reported questionnaires in 10 out of the 12 studies included [20–22, 25–31]. We classified these outcomes according to the four main categories pre-determined in our eligibility criteria (see Table 1). Indeed, family physician health status was examined as an outcome in two studies [28, 31], family physician competence in one study [23], family physician psychosocial outcomes in four studies [21, 24, 26, 30], and family physician performance in five studies [20, 22, 25, 27, 29].

### Description of the risk of bias assessment

**Fig. 2:**
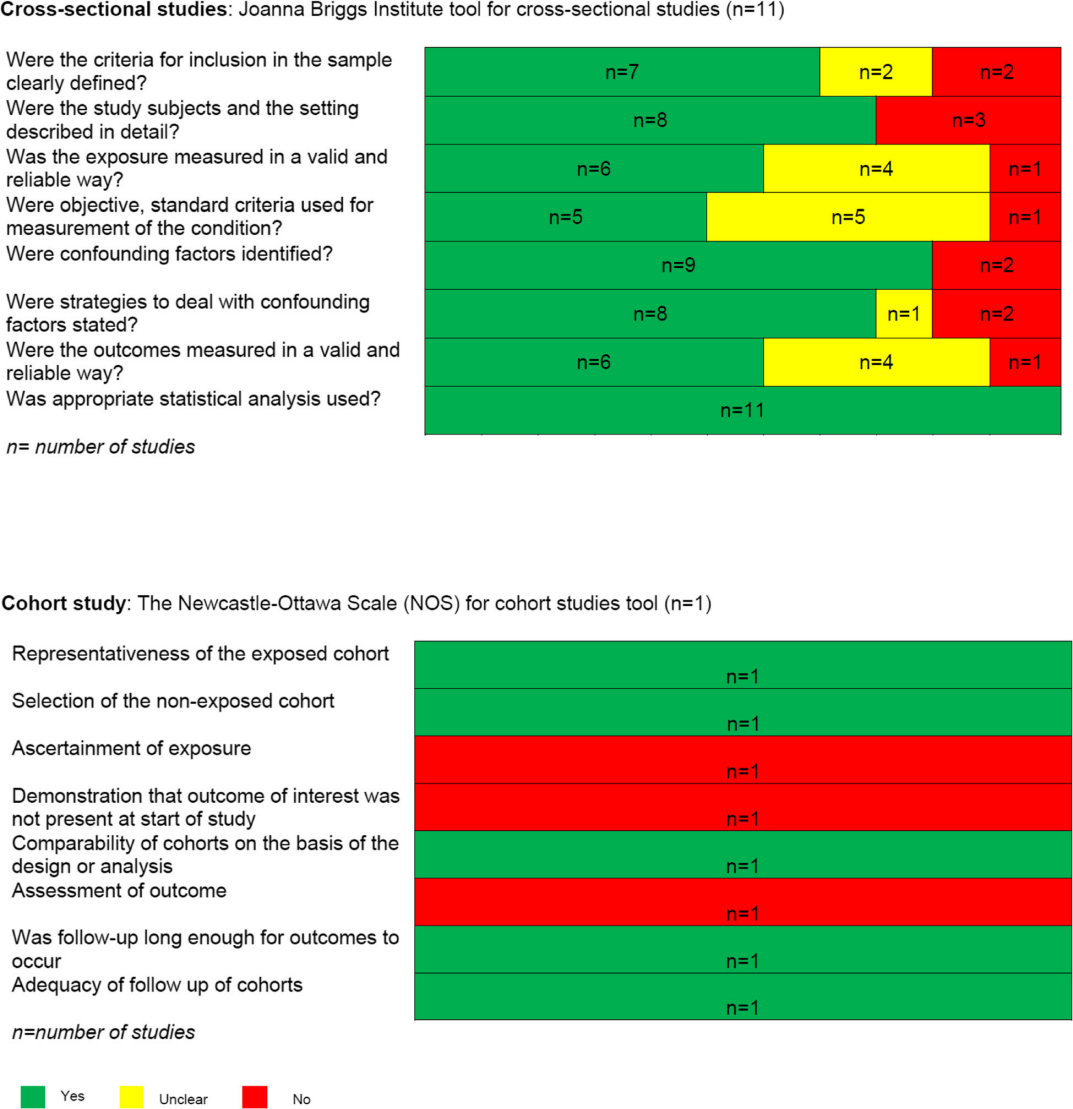
Assessment of risk of bias in studies included

Figure 2 presents the details of the risk of bias assessment in the included studies. For the cohort study, five out of eight assessment criteria were rated “yes” and the three others “no” [25]. For the cross-sectional studies, all assessment criteria were rated “yes” in the majority of studies, with a frequency varying from 5/11 for the criterion “*Was the exposure measured in a valid and reliable way?*” to 11/11 for the criterion *“Was appropriate statistical analysis used?*”

### Association between the scope of practice and family physician outcomes

The details of associations studied were reported in the Table 1 including the name, measurement and category for each variant of the scope of practice and, for each variant of family physician outcomes. We have also reported the crude and adjusted effects, their 95% confidence intervals and *p*-values when available.

### Scope of practice and family physician health status

This association was examined in two study [28, 31]. Indeed, a significant protective effect against burnout was observed when the clinical practice extended to inpatient medicine (odds ratio = 0.70, 95% CI 0.56–0.87) or to obstetric practice (odds ratio = 0.64, 95% CI 0.47–0.88) among new family physicians [28]. In contrast, the scope of practice measured with the Scope of Practice for Primary Care (SP4PC) Score is not associated with burnout in family physicians [31].

### Scope of practice and family physician competences

This association was also explored in one study [23]. Peterson et al. observed that the scope of practice assessed by the variety of clinical practices with a score ranging from zero to 30 seemed to be positively associated with the maintenance of family physician certification both in urban areas (odds ratio = 1.08, 95% CI 1.06–1.11) and in rural areas (odds ratio = 1.11, 95% CI 1.07–1.16).

### Scope of practice and family physician psychosocial outcomes

This association was explored in four studies [21, 24, 26, 30]. Eliason et al. showed that family physicians who routinely performed practice obstetrics seem to substantially improve their self-transcendence (e.g., universalism, *p*-value = 0.02) and reduce their self-enhancement (e.g., hedonism, *p*-value = 0.02), with adjustments made for sex and age [21]. In the same study, the authors showed that there were no associations between the inclusion of inpatient care in routine practice or the intensive care unit or critical care unit and family physician outcomes (e.g., desire to maintain customs of culture and religion or motivation to enhance and protect all people) [21]. Rivet et al. showed that numerous clinical procedures were associated with an increase in overall job satisfaction among family physicians (*p* = 0.0001) [24]. This same outcome was also enhanced among family physicians when they taught [24, 26]. Finally, the loss of meaning in work was reduced when family physicians taught but increased with they had administrative overload [30].

### Scope of practice and family physician performance

This association was explored in five studies [20, 22, 25, 27, 29]. The variety of practice settings (e.g., hospital and primary care center versus primary care center: 0.76, 95% CI 0.39–1.49) did not seem to be associated with family physician performance defined by the quality of antibiotic prescribing [25]. However, when the practice setting was more specific, for example, active hospital appointment, it seems to be associated with family physician performance defined by the score for the management of patients with multiple conditions (mean difference = 0.08 with a standard error of 0.036) [29]. The variety of clinical practices (e.g., walking in the clinic or emergency practices) did not seem to be associated with family physician performance [25, 27, 29]. In contrast, the variety of clinical procedures, mainly gynecological procedures, seemed to be associated with family physician performance [22] but not when the variety of clinical procedures was defined as performing deliveries in addition to family physician practices [20].

### Evaluation for the quality of cumulative evidence

The results of the quality of cumulative evidence were presented with details in the Additional file 2. It was not possible to combine the association measures because of the high heterogeneity of independent variables and outcomes identified and their measurement. Therefore, we evaluated each unique association studied. Moreover, no score was upgraded because of the exploratory nature of statistical analyses performed in the studies included. Considering the ratings of the risk of bias, inconsistency, indirectness, imprecision, or publication bias, the evidence available on the associations identified was of very low level.

## Discussion

### Summary of evidence

We identified a large variety in the scope of practice and family physician outcomes from a small number of included studies with a generally acceptable methodological quality. We found the following: 1) family physicians with diverse clinical and nonclinical activities seem to improve their psychosocial outcomes, mainly job satisfaction; 2) family physicians with a variety of clinical practices seem to improve their competences and health status compared to those who do not have a variety of clinical practices; 3) family physicians who perform clinical procedures (mainly extended to gynecological procedures) seem to have improved psychosocial outcomes (e.g., job satisfaction) compared to those who do not; and 4) some associations are not statistically significant. However, these results must be interpreted with caution because of the very low level of available evidence. Our findings led us to follow explanatory hypotheses.

### Explanatory hypotheses supported by literature

Family physicians with diverse activities seem to improve their psychosocial outcomes, mainly their job satisfaction. We could explain this result by the fact that the combination of two or more activities, such as clinical practices, teaching, research, or mentoring, could break the routine in daily work and increase the motivation of family physicians in their work [9, 10]. This could also be attractive for the profession of family medicine. For example, Lee et al. showed that the desire for a varied scope of practice is a predictor of medical students choosing a family medicine career [32].

Family physicians with a variety of clinical practice areas seem to improve their competences and health status compared to those who do not have a variety of clinical practice areas. The improvement in health status could be explained by family physician satisfaction related to work life, which represents the fourth goal of the quadruple aim [33]. The competence improvement could be explained by the fact that family physicians perform a variety of clinical practices compared to others who do not need additional clinical training and practice. For example, Basilious et al. showed in a sample of 110 family physicians that 30% lacked knowledge of glaucoma medications and 57% lacked knowledge of their side effects [34]. These results suggest that family physicians who consult patients diagnosed with glaucoma could benefit from educational materials to improve their knowledge. This could contribute to reinforcing and enhancing the scope of their competences. Another explanation for the competence improvement could be the fact that the certifying examination is based on the breadth of the specialty. So a family physician whose clinical practice is broader, thus knowledge matching more closely the content of the exam, will have better exam performance due to being actively involved clinical care across the breadth of the specialty.

Family physicians who also perform clinical procedures (mainly extended to gynecological procedures) seem to have improved psychosocial outcomes (e.g., job satisfaction) compared to those who do not. This result could be explained by the fact that gynecological procedures are known as an opportunity to care for a healthy population. For example, Al shalehi et al. showed that one of the main factors attracting both men and women medical students to the obstetrical/gynecological specialty was the opportunity to care for a healthy population [35]. Therefore, performing gynecological procedures could generate an improvement of psychosocial outcomes as well as job satisfaction in family physicians. Another explanation for the job satisfaction could be the fact that the practice of large clinical procedures by family physicians breaks the routine of standard care and uses a different part of their brain. This could lead them to higher job satisfaction.

Some associations are not statistically significant. These results may be partially explained by one main factor. Indeed, most of the studies included were cross-sectional and explored the associations between different scopes of practice and family physician outcomes. For these studies, authors did not estimate a sufficient sample size necessary to determine a significant effect for a specific association. As shown by Charan and Biswas, effect size is not considered in the calculation of sample size for cross-sectional studies [36]. Therefore, the effects may have lacked statistical significance due to insufficient statistical power.

### Limitations and strengths

The main limitation of the present review is the very small number of studies identified per association explored. This could have a few consequences on our results. First, it limited us in the data analysis. For example, it was not possible to quantitatively pool the effects due to study heterogeneity. Second, our results need to be confirmed by future research because of very low level of available evidence. For example, more studies on a given association would have permitted us to pool the effects to increase the statistical power and obtain either a significant effect or more precision to determine a real absence of an effect. Third, our results are less generalizable. Indeed, the number of settings studied was very small with a limited diversity, and the populations studied did not seem guarantee a representativity. Another limitation is the fact that most studies included were cross-sectional (91,7%). This makes it difficult to establish a causal link between variables of interest. In fact, we cannot determine if the scope of practice is the main cause of the family physician outcomes identified. Future research based on cohort studies or experimental trials with rigorous methodology could help to reduce this gap.

However, this review has a few strengths. First, we used a rigorous methodology to perform the different steps of the review; thus, our results are both comprehensive and reproducible. Second, we consulted and involved a content expert in the review process. Indeed, her clinical management expertise helped us to better define the scope of practice and to better identify relevant articles. Therefore, our results have the potential to be relevant and useful to knowledge users such as medical students, family physicians, and healthcare system managers.

## Conclusions

Although the consulted literature is scattered and includes a small number of relevant studies, we found that the scope of practice improves some family physician outcomes but with a very low level of evidence available. We also succeeded in listing and categorizing different scopes of practice and family physician outcomes. Our findings could be useful not only to monitor the scope of practice in family medicine but also to generate numerous interesting research hypotheses. For knowledge users such as clinicians, they could use our findings to determine relevant associations that need to be studied in greater depth. For researchers, the literature studied remains exploratory and is not yet very convincing. Therefore, it would be important to design and implement studies with rigorous methodologies to better treat these issues. Conscious of these gaps, health system policy-makers could encourage these future research.

## Supplementary Information

**Additional file 1.** Search strategy.

**Additional file 2.** Summary of findings.

## Data Availability

All data and materials used during the present systematic review are available from the corresponding author.

## Abbreviations

CI: Confidence Interval
PICOS: Population, Intervention/Exposure, Outcome, Study design
PRISMA: Preferred Reporting Items for Systematic Reviews and Meta-Analyses
USA: United States of America
