# Supplementary material for: Effects of the scope of practice on family physicians: a systematic review
Source: BMC Fam Pract. 2021 Jan 8;22:12. doi: 10.1186/s12875-020-01328-1 (PMC7796628; doi:10.1186/s12875-020-01328-1)
Supplement: Supplementary file 1 — Additional file 1. Search strategy. [file 12875_2020_1328_MOESM1_ESM.docx]

**Appendix 1**

Family Medicine

Final Strategies

***************************

*Primary Studies*

Database: Ovid MEDLINE: Epub Ahead of Print, In-Process & Other Non-Indexed Citations, Ovid **MEDLINE® Daily and Ovid MEDLINE® <1946-Present>,**

**2020-11-10**

Search Strategy:

--------------------------------------------------------------------------------

1 Family Practice/ (65326)

2 General Practice/ (13526)

3 Physicians, Family/ (16444)

4 Physicians, Primary Care/ (3571)

5 (family doctor? or family medicine or family practice? or family physician? or family practitioner?).tw,kf. (35673)

6 (general practice? or general physician? or general practitioner?).tw,kf. (84789)

7 ((doctor? or physician? or practitioner? or FP or GP) adj3 primary care).tw,kf. (25140)

8 or/1-7 [FAMILY/PRIMARY CARE PHYSICIANS/SETTINGS] (173200)

9 Practice Patterns, Physicians'/ (60959)

10 practice pattern?.tw,kf. (9092)

11 (scope? adj3 practice?).tw,kf. (4256)

12 Family Practice/og [organization & administration] (5327)

13 Family Practice/st [standards] (5254)

14 Family Practice/td [trends] (2425)

15 General Practice/og [organization & administration] (1197)

16 General Practice/st [standards] (1032)

17 General Practice/td [trends] (436)

18 Physicians, Family/og [organization & administration] (372)

19 Physicians, Family/st [standards] (907)

20 Physicians, Family/td [trends] (276)

21 Physicians, Primary Care/og [organization & administration] (167)

22 Physicians, Primary Care/st [standards] (288)

23 Physicians, Primary Care/td [trends] (127)

24 exp Primary Health Care/og [organization & administration] (27197)

25 exp Primary Health Care/st [standards] (16250)

26 exp Primary Health Care/td [trends] (5798)

27 exp Delivery of Health Care/og [organization & administration] (62981)

28 exp Delivery of Health Care/st [standards] (37519)

29 exp Delivery of Health Care/td [trends] (41715)

30 ((broad$2 or comprehensive* or diverse or diversif* or diversit* or extend$2 or extensive* or full service? or pluralit* or variable or varied or versatil* or wide$2) adj3 (practi#e? or practi#ing)).tw,kf. (12139)

31 ((broad$2 or comprehensive* or diverse or diversif* or diversit* or extend$2 or extensive* or pluralit* or variable or varied or versatil* or wide$2) adj3 scope?).tw,kf. (8708)

32 or/9-31 [SCOPE OF PRACTICE] (250193)

33 8 and 32 [FAMILY PHYSICIANS - SCOPE OF PRACTICE] (32638)

34 Clinical Competence/ (94434)

35 (clinical* adj3 (skill* or competen*)).tw,kf. (14237)

36 (clinical* adj3 knowledge).tw,kf. (12202)

37 (clinical* adj3 perform*).tw,kf. (41434)

38 Cultural Competency/ (5654)

39 (cultural* adj3 (skill* or competen*)).tw,kf. (6161)

40 Work Performance/ (897)

41 ((career? or job? or occupation* or professional* or work*) adj3 perform*).tw,kf. (31605)

42 Burnout, Professional/ (12358)

43 burnout?.tw,kf. (12531)

44 burn* out?.tw,kf. (1753)

45 Occupational Diseases/ (83684)

46 Occupational Stress/ (1812)

47 ((career? or job? or occupation* or professional* or work*) adj3 stress*).tw,kf. (16256)

48 Work-Life Balance/ (662)

49 (work adj1 life adj3 (balanc* or harmony)).tw,kf. (1420)

50 Job Satisfaction/ (25203)

51 ((career? or job? or occupation* or professional* or work*) adj3 satisf*).tw,kf. (15238)

52 exp Physicians/px [psychology] (24441)

53 ((doctor? or physician? or practitioner? or FP or GP) adj3 (wellbeing or well-being)).tw,kf. (755)

54 exp Adaptation, Psychological/ (128279)

55 (adapt* adj3 (behav* or psycholog*)).tw,kf. (13037)

56 Quality of Life/ (199419)

57 ((doctor? or physician? or practitioner? or FP or GP) adj3 (quality adj2 life)).tw,kf. (536)

58 Absenteeism/ (9147)

59 absentee*.tw,kf. (6377)

60 Presenteeism/ (332)

61 presentee*.tw,kf. (1527)

62 productivit*.tw,kf. (61974)

63 ((work* or employ*) adj3 (absenc* or absent* or presenc* or present*)).tw,kf. (109964)

64 ((work* or employ*) adj3 abilit*).tw,kf. (7793)

65 (time adj1 away).tw,kf. (688)

66 Sick Leave/ (5908)

67 (medical leave? or sick leave?).tw,kf. (5726)

68 Personal Autonomy/ (17196)

69 ((autonom* or freedom) adj3 (doctor? or physician? or practitioner? or FP or GP or person$2 or personally or self)).tw,kf. (4380)

70 Self Concept/ (57124)

71 (self adj2 (concept* or conceiv* or perception? or perceiv* or esteem*)).tw,kf. (44369)

72 Attitude of Health Personnel/ (123098)

73 (attitud* adj3 (doctor? or physician? or practitioner? or FP or GP)).tw,kf. (6371)

74 or/34-73 [PHYSICIAN OUTCOMES] (1011462)

75 33 and 74 [FAMILY PHYSICIANS - SCOPE OF PRACTICE - OUTCOMES] (8378)

76 (controlled clinical trial or randomized controlled trial).pt. (605700)

77 clinical trials as topic.sh. (193603)

78 Randomized Controlled Trials as Topic/ (137822)

79 (randomi#ed or randomi#ation? or randomly or RCT? or placebo*).tw,kf. (1015906)

80 ((singl* or doubl* or trebl* or tripl*) adj (mask* or blind* or dumm*)).tw,kf. (175833)

81 trial.ti. (228357)

82 or/76-81 (1444778)

83 75 and 82 [RCTs] (822)

84 controlled clinical trial.pt. (93916)

85 Controlled Clinical Trial/ or Controlled Clinical Trials as Topic/ (99419)

86 (control* adj2 trial*).tw,kf. (286656)

87 Non-Randomized Controlled Trials as Topic/ (796)

88 (nonrandom* or non-random* or quasi-random* or quasi-experiment*).tw,kf. (58491)

89 (nRCT or nRCTs or non-RCT?).tw,kf. (961)

90 Controlled Before-After Studies/ (561)

91 (control* adj3 ("before and after" or "before after")).tw,kf. (4447)

92 Interrupted Time Series Analysis/ (1032)

93 time series.tw,kf. (32678)

94 (pre- adj3 post-).tw,kf. (86465)

95 (pretest adj3 posttest).tw,kf. (5778)

96 Historically Controlled Study/ (191)

97 (control* adj2 stud$3).tw,kf. (239059)

98 Control Groups/ (1704)

99 (control* adj2 group?).tw,kf. (516968)

100 or/84-99 (1173564)

101 75 and 100 [NON-RCTs] (533)

102 exp Cohort Studies/ (2051717)

103 cohort?.tw,kf. (619435)

104 Retrospective Studies/ (849872)

105 (longitudinal or prospective or retrospective).tw,kf. (1318683)

106 ((followup or follow-up) adj (study or studies)).tw,kf. (52280)

107 Observational study.pt. (87619)

108 (observation$2 adj (study or studies)).tw,kf. (114981)

109 ((population or population-based) adj (study or studies or analys#s)).tw,kf. (17894)

110 ((multidimensional or multi-dimensional) adj (study or studies)).tw,kf. (114)

111 Comparative Study.pt. (1874829)

112 ((comparative or comparison) adj (study or studies)).tw,kf. (116784)

113 exp Case-Control Studies/ (1116919)

114 ((case-control* or case-based or case-comparison) adj (study or studies)).tw,kf. (110313)

115 or/102-114 (4595636)

116 75 and 115 [OBSERVATIONAL STUDIES] (1551)

117 Cross-Sectional Studies/ (342704)

118 (cross-section* or crosssection*).tw,kf. (409101)

119 "Surveys and Questionnaires"/ (473927)

120 Health Surveys/ (63213)

121 survey*.tw,kf. (667143)

122 questionnaire*.tw,kf. (1528154)

123 or/117-122 (1570474)

124 75 and 123 [CROSS-SECTIONAL STUDIES/SURVEYS/QUESTIONNAIRES] (4167)

125 83 or 101 or 116 or 124 (5045)

126 exp Animals/ not (exp Animals/ and Humans/) (4754125)

127 125 not 126 [ANIMAL-ONLY REMOVED] (5045)

128 (comment or editorial or news or newspaper article).pt. (1457148)

129 127 not 128 [OPINION PIECES REMOVED] (5008)

130 limit 129 to yr="2018 -Current" (459)

**Embase-Elsevier (2020-11-12)**

1 'general practice'/de (85259)

2 'general practitioner'/de (101287)

3 "family doctor$":ti,ab,kw OR "family medicine":ti,ab,kw OR "family practice$":ti,ab,kw OR "family physician$":ti,ab,kw OR "family practitioner$":ti,ab,kw (46223)

4 "general practice$":ti,ab,kw OR "general physician$":ti,ab,kw OR "general practitioner$":ti,ab,kw (111068)

5 ((doctor$ OR physician$ OR practitioner$ OR Ip OR gp) NEAR/3 'primary care'):ti,ab,kw (35483)

6 #1 OR #2 OR #3 OR #4 OR #5 [FAMILY PHYSICIANS] (249128)

7 'clinical practice'/de (292475)

8 "practice pattern$":ti,ab,kw (14606)

9 (scope$ NEAR/3 practice$):ti,ab,kw (5417)

10 ((broad* OR comprehensive* OR diverse OR diversif* OR diversit* OR extend* OR extensive* OR "full service$" OR pluralit* OR variable OR varied OR versatiI* OR wide*) NEAR/3 (practi?e$ OR practi?ing)):ti,ab,kw (18901)

11 ((broad* OR comprehensive8 OR diverse OR diversif* OR diversit* OR extend* OR extensive* OR pluralit* OR variable OR varied OR versatiI* OR wide*) NEAR/3 scope$):ti,ab,kw (11963)

12 #7 OR #8 OR #9 OR #10 OR #11 [SCOPE OF PRACTICE] (335401)

13 #6 and #12 [FAMILY PHYSICIANS - SCOPE OF PRACTICE] (17436)

14 'clinical competence'/de (61030)

15 (clinical* NEAR/3 (skill* OR competen*)):ti,ab,kw (19362)

16 (clinical* NEAR/3 knowledge):ti,ab,kw (16867)

17 (clinical* NEAR/3 perform*):ti,ab,kw (62404)

18 'cultural competence'/de (6667)

19 (cultural* NEAR/3 (skill* OR competen*)):ti,ab,kw (7431)

20 'job performance'/de (16879)

21 ((career? or job? or occupation* or professional* or work*) NEAR/3 perform*):ti,ab,kw (41695)

22 'burnout'/de (18559)

23 burnout$:ti,ab,kw (16820)

24 "burn* out$":ti,ab,kw (2661)

25 'occupational disease'/de (137320)

26 'job stress'/de (7283)

27 ((career$ OR job$ OR occupation* OR professional* OR work*) NEAR/3 stress*):ti,ab,kw (30364)

28 'work-life balance'/de (1488)

29 (work NEAR/1 life NEAR/3 (balanc* OR harmony)):ti,ab,kw (1908)

30 'job satisfaction'/de (30680)

31 ((career$ OR job$ OR occupation* OR professional* OR work*) NEAR/3 satisf*):ti,ab,kw (18852)

32 ((doctor$ OR physician$ OR practitioner$ OR fp OR gp) NEAR/3 (wellbeing OR 'well being')):ti,ab,kw (962)

33 'coping behavior'/de (60237)

34 (adapt* NEAR/3 (behav* OR psycholog*)):ti,ab,kw (15493)

35 'quality of working life'/de (524)

36 ((doctor$ OR physician$ OR practitioner$ OR fp OR gp) NEAR/3 ('quality of life' OR 'quality on life' OR 'quality work life' OR 'life quality' OR 'quality-adjusted life' OR 'quality life' OR 'quality and life')):ti,ab,kw (841)

37 'absenteeism'/de (18426)

38 absentee*:ti,ab,kw (9322)

39 'presenteeism'/de (1406)

40 presentee*:ti,ab,kw (2440)

41 'productivity'/de (41632)

42 productivit*:ti,ab,kw (75005)

43 ((work* OR employ*) NEAR/3 (absenc* OR absent* OR presenc* OR present*)):ti,ab,kw (136297)

44 ((work* OR employ*) NEAR/3 abilit*):ti,ab,kw (10580)

45 (time NEAR/1 away):ti,ab,kw (999)

46 'medical leave'/de (6906)

47 "medical leave$":ti,ab,kw OR "sick leave$":ti,ab,kw (7473)

48 'personal autonomy'/de (13766)

49 ((autonom* OR freedom) NEAR/3 (doctor$ OR physician$ OR practitioner$ OR fp OR gp OR person* OR personally OR self)):ti,ab,kw (5514)

50 'self concept'/de (94464)

51 (self NEAR/2 (concept* OR conceiv* OR perception$ OR perceiv* OR esteem*)):ti,ab,kw (55444)

52 'professional image'/de (513)

53 'physician attitude'/de (53921)

54 (attitud* NEAR/3 (doctor$ OR physician$ OR practitioner$ OR fp OR gp)):ti,ab,kw (8181)

55 #14 OR #15 OR #16 OR #17 OR #18 OR #19 OR #20 OR #21 OR #22 OR #23 OR #24 OR #25 OR #26 OR #27 OR #28 OR #29 OR #30 OR #31 OR #32 OR #33 OR #34 OR #35 OR #36 OR #37 OR #38 OR #39 OR #40 OR #41 OR #42 OR #43 OR #44 OR #45 OR #46 OR #47 OR #48 OR #49 OR #50 OR #51 OR #52 OR #53 OR #54 [PHYSICIAN OUTCOMES] (842905)

56 #13 AND #55 [FAMILY PHYSICIANS - SCOPE OF PRACTICE - OUTCOMES] (3190)

57 'randomized controlled trial'/de (628557)

58 'controlled clinical trial'/de (431601)

59 'clinical trial (topic)'/exp (336595)

60 (randomi?ed or randomi?ation$ or randomly or RCT$ or placebo*):ti,ab,kw (1427160)

61 ((singl* or doubl* or trebl* or tripl*) NEAR/0 (mask* or blind* or dumm*)):ti,ab,kw (49)

62 trial:ti (318784)

63 #57 OR #58 OR #59 OR #60 OR #61 OR #62 (1967043)

64 #56 AND #63 [RCTs] (302)

65 "controlled clinical trial":it (0)

66 'controlled clinical trial (topic)'/exp (197497)

67 (control* NEAR/2 trial*):ti,ab,kw (381409)

68 (nonrandom* or non-random* or quasi-random* or quasi-experiment*):ti,ab,kw (74844)

69 (nRCT or nRCTs or "non-RCT$"):ti,ab,kw (1378)

70 (control* NEAR/3 ("before and after" or "before after")):ti,ab,kw (6209)

71 'time series analysis'/de (27246)

72 "time series":ti,ab,kw (37488)

73 'pretest posttest control group design'/de (512)

74 (pre- NEAR/3 post-):ti,ab,kw (4634)

75 (pretest NEAR/3 posttest):ti,ab,kw (10761)

76 'controlled study'/de (7784234)

77 (control* NEAR/2 stud*):ti,ab,kw (323585)

78 'control group'/de (108334)

79 (control* NEAR/2 group$):ti,ab,kw (741907)

80 #65 OR #66 OR #67 OR #68 OR #69 OR #70 OR #71 OR #72 OR #73 OR #74 OR #75 OR #76 OR #77 OR #78 OR #79 (8513501)

81 #56 AND #80 [NON-RCTs] (574)

82 'cohort analysis'/de (633121)

83 cohort?:ti,ab,kw (165353)

84 'retrospective study'/de (988165)

85 'longitudinal study'/de (146357)

86 'prospective study'/de (639200)

87 (longitudinal or prospective or retrospective):ti,ab,kw (2022392)

88 'follow up'/de (1617297)

89 ((followup or follow-up) NEAR/1 (study or studies)):ti,ab,kw (74072)

90 'observational study'/de (196506)

91 (observation* NEAR/1 (study or studies)):ti,ab,kw (212774)

92 'population research'/de (110569)

93 ((population or population-based) NEAR/1 (study or studies or analys?s)):ti,ab,kw (183832)

94 ((multidimensional or multi-dimensional) NEAR/1 (study or studies)):ti,ab,kw (189)

95 'comparative study'/exp (1461170)

96 ((comparative or comparison) NEAR/1 (study or studies)):ti,ab,kw (148001)

97 'case control study'/exp (179733)

98 ((case-control* or case-based or case-comparison) NEAR/1 (study or studies)):ti,ab,kw (144090)

99 #82 OR #83 OR #84 OR #85 OR #86 OR #87 OR #88 OR #89 OR #90 OR #91 OR #92 OR #93 OR #94 OR #95 OR #96 OR #97 OR #98 (5585067)

100 #56 AND #99 [OBSERVATIONAL STUDIES] (445)

101 'cross-sectional study'/de (374746)

102 (cross-section* or crosssection*):ti,ab,kw (529979)

103 'questionnaire'/exp (731104)

104 'health survey'/de (200050)

105 survey*:ti,ab,kw (861788)

106 questionnaire*:ti,ab,kw (768635)

107 #101 OR #102 OR #103 OR #104 OR #105 OR #106 (2052530)

108 #56 AND #107 [CROSS-SECTIONAL STUDIES/SURVEYS/QUESTIONNAIRES] (1413)

109 #64 OR #81 OR #100 OR #108 [ALL STUDY DESIGNS] (1799)

110 'animal'/exp or 'animal model'/exp or 'animal experiment'/exp or 'nonhuman'/de or 'vertebrate'/exp (29777624)

111 'human'/exp or 'human experiment'/exp (2263515)

112 #110 not #111 (7143600)

113 #109 not #112 [ANIMAL-ONLY REMOVED] (1797)

114 editorial:it (665412)

115 letter:it not (letter:it and 'randomized controlled trial'/de) (1126246)

116 #113 not (#114 or #115) [OPINION PIECES REMOVED] (1765)

117 conference abstract:it (3887154)

118 #116 not #117 [CONFERENCE ABSTRACTS REMOVED] (1908)

119 #116 NOT #117 AND [2018-2020]/py (201)

***************************

**ERIC-Proquest 2020-11-11**

| Set# | Searched for | Databases | Results |
| --- | --- | --- | --- |
| S1 | su(Family Practice (Medicine)) | ERIC | 599 |
| S2 | ti(family PRE/0 doctor? OR family PRE/0 medicine OR family PRE/0 practice? OR family PRE/0 physician? OR family PRE/0 practitioner?) OR ab(family PRE/0 doctor? OR family PRE/0 medicine OR family PRE/0 practice? OR family PRE/0 physician? OR family PRE/0 practitioner?) | ERIC | 907 |
| S3 | ti(general P/0 practice? OR general P/0 physician? OR general P/0 practitioner?) OR ab(general P/0 practice? OR general P/0 physician? OR general P/0 practitioner?) | ERIC | 456 |
| S4 | ti((doctor? N/3 "primary care") or (physician? N/3 "primary care") or (practitioner? N/3 "primary care") or (FP N/3 "primary care") or (GP N/3 "primary care")) OR ab((doctor? N/3 "primary care") or (physician? N/3 "primary care") or (practitioner? N/3 "primary care") or (FP N/3 "primary care") or (GP N/3 "primary care")) | ERIC | 334 |
| S5 | su(Family Practice (Medicine)) OR (ti(family PRE/0 doctor? OR family PRE/0 medicine OR family PRE/0 practice? OR family PRE/0 physician? OR family PRE/0 practitioner?) OR ab(family PRE/0 doctor? OR family PRE/0 medicine OR family PRE/0 practice? OR family PRE/0 physician? OR family PRE/0 practitioner?)) OR (ti(general P/0 practice? OR general P/0 physician? OR general P/0 practitioner?) OR ab(general P/0 practice? OR general P/0 physician? OR general P/0 practitioner?)) OR (ti((doctor? N/3 "primary care") or (physician? N/3 "primary care") or (practitioner? N/3 "primary care") or (FP N/3 "primary care") or (GP N/3 "primary care")) OR ab((doctor? N/3 "primary care") or (physician? N/3 "primary care") or (practitioner? N/3 "primary care") or (FP N/3 "primary care") or (GP N/3 "primary care"))) | ERIC  These databases are searched for part of your query. | 1745 |
| S6 | ti(practice P/0 pattern?) OR ab(practice P/0 pattern?) | ERIC | 109 |
| S7 | ti(scope? n/3 practice?) OR ab(scope? n/3 practice?) | ERIC | 227 |
| S8 | ti((broad* NEAR/3 scope? OR comprehensive* NEAR/3 scope? OR diverse NEAR/3 scope? OR diversif* NEAR/3 scope? OR diversit* NEAR/3 scope? OR extend* NEAR/3 scope? OR extensive* NEAR/3 scope? OR full-service? NEAR/3 scope? OR pluralit* NEAR/3 scope? OR variable NEAR/3 scope? OR varied NEAR/3 scope? OR versatil* NEAR/3 scope? OR wide NEAR/3 scope? OR widen NEAR/3 scope? OR widely NEAR/3 scope?)) OR ab((broad* NEAR/3 scope? OR comprehensive* NEAR/3 scope? OR diverse NEAR/3 scope? OR diversif* NEAR/3 scope? OR diversit* NEAR/3 scope? OR extend* NEAR/3 scope? OR extensive* NEAR/3 scope? OR full-service? NEAR/3 scope? OR pluralit* NEAR/3 scope? OR variable NEAR/3 scope? OR varied NEAR/3 scope? OR versatil* NEAR/3 scope? OR wide NEAR/3 scope? OR widen NEAR/3 scope? OR widely NEAR/3 scope?)) | ERIC | 1382 |
| S9 | (ti(practice P/0 pattern?) OR ab(practice P/0 pattern?)) OR (ti(scope? n/3 practice?) OR ab(scope? n/3 practice?)) OR (ti((broad* NEAR/3 scope? OR comprehensive* NEAR/3 scope? OR diverse NEAR/3 scope? OR diversif* NEAR/3 scope? OR diversit* NEAR/3 scope? OR extend* NEAR/3 scope? OR extensive* NEAR/3 scope? OR full-service? NEAR/3 scope? OR pluralit* NEAR/3 scope? OR variable NEAR/3 scope? OR varied NEAR/3 scope? OR versatil* NEAR/3 scope? OR wide NEAR/3 scope? OR widen NEAR/3 scope? OR widely NEAR/3 scope?)) OR ab((broad* NEAR/3 scope? OR comprehensive* NEAR/3 scope? OR diverse NEAR/3 scope? OR diversif* NEAR/3 scope? OR diversit* NEAR/3 scope? OR extend* NEAR/3 scope? OR extensive* NEAR/3 scope? OR full-service? NEAR/3 scope? OR pluralit* NEAR/3 scope? OR variable NEAR/3 scope? OR varied NEAR/3 scope? OR versatil* NEAR/3 scope? OR wide NEAR/3 scope? OR widen NEAR/3 scope? OR widely NEAR/3 scope?))) | ERIC  These databases are searched for part of your query. | 1690 |
| S10 | (su(Family Practice (Medicine)) OR (ti(family PRE/0 doctor? OR family PRE/0 medicine OR family PRE/0 practice? OR family PRE/0 physician? OR family PRE/0 practitioner?) OR ab(family PRE/0 doctor? OR family PRE/0 medicine OR family PRE/0 practice? OR family PRE/0 physician? OR family PRE/0 practitioner?)) OR (ti(general P/0 practice? OR general P/0 physician? OR general P/0 practitioner?) OR ab(general P/0 practice? OR general P/0 physician? OR general P/0 practitioner?)) OR (ti((doctor? N/3 "primary care") or (physician? N/3 "primary care") or (practitioner? N/3 "primary care") or (FP N/3 "primary care") or (GP N/3 "primary care")) OR ab((doctor? N/3 "primary care") or (physician? N/3 "primary care") or (practitioner? N/3 "primary care") or (FP N/3 "primary care") or (GP N/3 "primary care")))) AND ((ti(practice P/0 pattern?) OR ab(practice P/0 pattern?)) OR (ti(scope? n/3 practice?) OR ab(scope? n/3 practice?)) OR (ti((broad* NEAR/3 scope? OR comprehensive* NEAR/3 scope? OR diverse NEAR/3 scope? OR diversif* NEAR/3 scope? OR diversit* NEAR/3 scope? OR extend* NEAR/3 scope? OR extensive* NEAR/3 scope? OR full-service? NEAR/3 scope? OR pluralit* NEAR/3 scope? OR variable NEAR/3 scope? OR varied NEAR/3 scope? OR versatil* NEAR/3 scope? OR wide NEAR/3 scope? OR widen NEAR/3 scope? OR widely NEAR/3 scope?)) OR ab((broad* NEAR/3 scope? OR comprehensive* NEAR/3 scope? OR diverse NEAR/3 scope? OR diversif* NEAR/3 scope? OR diversit* NEAR/3 scope? OR extend* NEAR/3 scope? OR extensive* NEAR/3 scope? OR full-service? NEAR/3 scope? OR pluralit* NEAR/3 scope? OR variable NEAR/3 scope? OR varied NEAR/3 scope? OR versatil* NEAR/3 scope? OR wide NEAR/3 scope? OR widen NEAR/3 scope? OR widely NEAR/3 scope?)))) | ERIC  These databases are searched for part of your query. | 11 |

| **Section/topic** | **#** | **Checklist item** | **Reported on page #** |
| --- | --- | --- | --- |
| **TITLE** | | |  |
| Title | 1 | Identify the report as a systematic review, meta-analysis, or both. | **# 1** |
| **ABSTRACT** | | |  |
| Structured summary | 2 | Provide a structured summary including, as applicable: background; objectives; data sources; study eligibility criteria, participants, and interventions; study appraisal and synthesis methods; results; limitations; conclusions and implications of key findings; systematic review registration number. | **# 3-4** |
| **INTRODUCTION** | | |  |
| Rationale | 3 | Describe the rationale for the review in the context of what is already known. | **# 5-6** |
| Objectives | 4 | Provide an explicit statement of questions being addressed with reference to participants, interventions, comparisons, outcomes, and study design (PICOS). | **# 6** |
| **METHODS** | | |  |
| Protocol and registration | 5 | Indicate if a review protocol exists, if and where it can be accessed (e.g., Web address), and, if available, provide registration information including registration number. | **#6** |
| Eligibility criteria | 6 | Specify study characteristics (e.g., PICOS, length of follow-up) and report characteristics (e.g., years considered, language, publication status) used as criteria for eligibility, giving rationale. | **# 6-7** |
| Information sources | 7 | Describe all information sources (e.g., databases with dates of coverage, contact with study authors to identify additional studies) in the search and date last searched. | **# 7** |
| Search | 8 | Present full electronic search strategy for at least one database, including any limits used, such that it could be repeated. | **# 7** |
| Study selection | 9 | State the process for selecting studies (i.e., screening, eligibility, included in systematic review, and, if applicable, included in the meta-analysis). | **# 8** |
| Data collection process | 10 | Describe method of data extraction from reports (e.g., piloted forms, independently, in duplicate) and any processes for obtaining and confirming data from investigators. | **# 8** |
| Data items | 11 | List and define all variables for which data were sought (e.g., PICOS, funding sources) and any assumptions and simplifications made. | **# 8-9** |
| Risk of bias in individual studies | 12 | Describe methods used for assessing risk of bias of individual studies (including specification of whether this was done at the study or outcome level), and how this information is to be used in any data synthesis. | **# 9** |
| Summary measures | 13 | State the principal summary measures (e.g., risk ratio, difference in means). | **# 9** |
| Synthesis of results | 14 | Describe the methods of handling data and combining results of studies, if done, including measures of consistency (e.g., I^2^) for each meta-analysis. | **# 9** |
| Risk of bias across studies | 15 | Specify any assessment of risk of bias that may affect the cumulative evidence (e.g., publication bias, selective reporting within studies). | **# 9** |
| Additional analyses | 16 | Describe methods of additional analyses (e.g., sensitivity or subgroup analyses, meta-regression), if done, indicating which were pre-specified. | **# 9** |
| **RESULTS** | | |  |
| Study selection | 17 | Give numbers of studies screened, assessed for eligibility, and included in the review, with reasons for exclusions at each stage, ideally with a flow diagram. | **#10** |
| Study characteristics | 18 | For each study, present characteristics for which data were extracted (e.g., study size, PICOS, follow-up period) and provide the citations. | **# 10** |
| Risk of bias within studies | 19 | Present data on risk of bias of each study and, if available, any outcome level assessment (see item 12). | **# 11-12** |
| Results of individual studies | 20 | For all outcomes considered (benefits or harms), present, for each study: (a) simple summary data for each intervention group (b) effect estimates and confidence intervals, ideally with a forest plot. | **# 11-13** |
| Synthesis of results | 21 | Present results of each meta-analysis done, including confidence intervals and measures of consistency. | **Not applicable** |
| Risk of bias across studies | 22 | Present results of any assessment of risk of bias across studies (see Item 15). | **Not applicable** |
| Additional analysis | 23 | Give results of additional analyses, if done (e.g., sensitivity or subgroup analyses, meta-regression [see Item 16]). | **Not applicable** |
| **DISCUSSION** | | |  |
| Summary of evidence | 24 | Summarize the main findings including the strength of evidence for each main outcome; consider their relevance to key groups (e.g., healthcare providers, users, and policy makers). | **# 15** |
| Limitations | 25 | Discuss limitations at study and outcome level (e.g., risk of bias), and at review-level (e.g., incomplete retrieval of identified research, reporting bias). | **# 17-18** |
| Conclusions | 26 | Provide a general interpretation of the results in the context of other evidence, and implications for future research. | **# 18-19** |
| **FUNDING** | | |  |
| Funding | 27 | Describe sources of funding for the systematic review and other support (e.g., supply of data); role of funders for the systematic review. | **# 1** |
