# Supplementary material for: Effects of the scope of practice on family physicians: a systematic review
Source: BMC Fam Pract. 2021 Jan 8;22:12. doi: 10.1186/s12875-020-01328-1 (PMC7796628; doi:10.1186/s12875-020-01328-1)
Supplement: Supplementary file 2 — Additional file 2. Summary of findings. [file 12875_2020_1328_MOESM2_ESM.docx]

| **Appendix 2**.: Summary of findings | | | | | | | | | |
| --- | --- | --- | --- | --- | --- | --- | --- | --- | --- |
| **Effect of the scope of practice on family physician health status**  *Population: Family physicians*  *Exposures: Scope of practice*  *Outcomes: Family physician health status*  *Settings: Multiple clinical practice settings* | | | | | | | | | |
| Outcomes | Exposure | Study design | N of studies | Risk of bias | Inconsistency | Indirectness | Imprecision | Publication bias | Certainty*  (Overall score) |
| Burnout | In-patient medicine practice | Cross-sectional(2) | 1 | None (0) | None (0) | Serious (-1) | None(0) | None (0) | 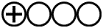  Very low |
| Burnout | Obstetric practices | Cross-sectional | 1 | None (0) | None (0) | Serious (-1) | None (0) | None (0) | 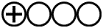  Very low |
| Burnout | Practice pediatric ambulatory care | Cross-sectional (2) | 1 | None (0) | None (0) | Serious (-1) | None (0) | None (0) | 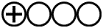  Very low |
| Burnout | Scope of practice | Cross-sectional (2) | 1 | None (0) | None (0) | None (0) | Serious (-1) | None (0) | 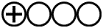  Very low |
|  |  |  |  |  |  |  |  |  |  |
| **Effect of the scope of practice on family physician competences**  *Population: Family physicians*  *Exposures: Scope of practice*  *Outcomes: Family physician competences*  *Settings: Urban area; Rural area* | | | | | | | | | |
| Outcomes | Exposure | Study design | N of studies | Risk of bias | Inconsistency | Indirectness | Imprecision | Publication bias | Overall score |
| Maintenance of certification | Clinical activities in urban area | Cross-sectional (2) | 1 | Borderline (-0.5) | None (0) | None (0) | None (0) | None (0) | 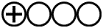  Very low |
| Maintenance of certification | Clinical activities in rural area | Cross-sectional (2) | 1 | Borderline (-0.5) | None (0) | None (0) | None (0) | None (0) | 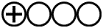  Very low |
|  |  |  |  |  |  |  |  |  |  |
| **Effect of the scope of practice on family physician psychosocial outcomes**  Population: Family physicians  Exposures: Scope of practice  Outcomes: Family physician psychosocial outcomes  Settings: Private offices or clinics, community clinics or health centres, or academic family medicine teaching units; all except free-standing walk-in clinics, nursing homes, hospital inpatient units, or emergency departments | | | | | | | | | |
| Outcomes | Exposure | Study design | N of studies | Risk of bias | Inconsistency | Indirectness | Imprecision | Publication bias | Overall score |
| Loss of meaning in work | Administrative overload | Cross-sectional (2) | 1 | Serious (-1) | None (0) | None (0) | None (0) | None (0) | 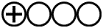  Very low |
| Loss of meaning in work | Teaching activities | Cross-sectional (2) | 1 | Serious (-1) | None (0) | None (0) | None (0) | None (0) | 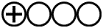  Very low |
| Overall job satisfaction | Teaching activities | Cross-sectional (2) | 1 | None (0) | None (0) | None (0) | None (0) | None (0) | 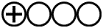  Very low |
| Overall job satisfaction | Variety of procedures done | Cross-sectional (2) | 1 | None (0) | None (0) | None (0) | None (0) | None (0) | 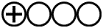  Very low |
| Overall job satisfaction | Teaching activities | Cross-sectional (2) | 1 | None (0) | None (0) | None (0) | None (0) | None (0) | 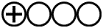  Very low |
| Security (personal value) | Inpatient care practices | Cross-sectional (2) | 1 | Serious (-1) | None (0) | Serious (-1) | Serious (-1) | None (0) | 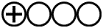  Very low |
| Hedonism (personal value) | Inpatient care practices | Cross-sectional (2) | 1 | Serious (-1) | None (0) | Serious (-1) | Serious (-1) | None (0) | 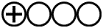  Very low |
| Universalism (personal value) | Inpatient care practices | Cross-sectional (2) | 1 | Serious (-1) | None (0) | Serious (-1) | Serious (-1) | None (0) | 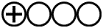  Very low |
| Conformity (personal value) | Inpatient care practices | Cross-sectional (2) | 1 | Serious (-1) | None (0) | Serious (-1) | Serious (-1) | None (0) | 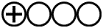  Very low |
| Power (personal value) | Inpatient care practices | Cross-sectional (2) | 1 | Serious (-1) | None (0) | Serious (-1) | None (1) | None (0) | 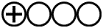  Very low |
| Benevolence (personal value) | Inpatient care practices | Cross-sectional (2) | 1 | Serious (-1) | None (0) | Serious (-1) | Serious (-1) | None (0) | 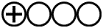  Very low |
| Self-direction (personal value) | Inpatient care practices | Cross-sectional (2) | 1 | Serious (-1) | None (0) | Serious (-1) | Serious (-1) | None (0) | 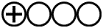  Very low |
| Stimulation (personal value) | Inpatient care practices | Cross-sectional (2) | 1 | Serious (-1) | None (0) | Serious (-1) | Serious (-1) | None (0) | 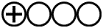  Very low |
| Achievement (personal value) | Inpatient care practices | Cross-sectional (2) | 1 | Serious (-1) | None (0) | Serious (-1) | Serious (-1) | None (0) | 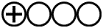  Very low |
| Tradition (personal value) | Inpatient care practices | Cross-sectional (2) | 1 | Serious (-1) | None (0) | Serious (-1) | Serious (-1) | None (0) | 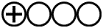  Very low |
| Security (personal value) | Inpatient care practices | Cross-sectional (2) | 1 | Serious (-1) | None (0) | Serious (-1) | Serious (-1) | None (0) | 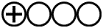  Very low |
| Hedonism (personal value) | Teaching medical trainees | Cross-sectional (2) | 1 | Serious (-1) | None (0) | Serious (-1) | None (0) | None (0) | 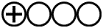  Very low |
| Universalism (personal value) | Teaching medical trainees | Cross-sectional (2) | 1 | Serious (-1) | None (0) | Serious (-1) | Serious (-1) | None (0) | 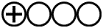  Very low |
| Conformity (personal value) | Teaching medical trainees | Cross-sectional (2) | 1 | Serious (-1) | None (0) | Serious (-1) | Serious (-1) | None (0) | 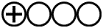  Very low |
| Power (personal value) | Teaching medical trainees | Cross-sectional (2) | 1 | Serious (-1) | None (0) | Serious (-1) | Serious (-1) | None (0) | 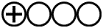  Very low |
| Benevolence (personal value) | Teaching medical trainees | Cross-sectional (2) | 1 | Serious (-1) | None (0) | Serious (-1) | Serious (-1) | None (0) | 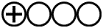  Very low |
| Self-direction (personal value) | Teaching medical trainees | Cross-sectional (2) | 1 | Serious (-1) | None (0) | Serious (-1) | Serious (-1) | None (0) | 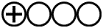  Very low |
| Stimulation (personal value) | Teaching medical trainees | Cross-sectional (2) | 1 | Serious (-1) | None (0) | Serious (-1) | Serious (-1) | None (0) | 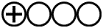  Very low |
| Achievement (personal value) | Teaching medical trainees | Cross-sectional (2) | 1 | Serious (-1) | None (0) | Serious (-1) | Serious (-1) | None (0) | 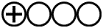  Very low |
| Tradition (personal value) | Teaching medical trainees | Cross-sectional (2) | 1 | Serious (-1) | None (0) | Serious (-1) | Serious (-1) | None (0) | 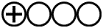  Very low |
| Security (personal value) | Teaching medical trainees | Cross-sectional (2) | 1 | Serious (-1) | None (0) | Serious (-1) | None(0) | None (0) | 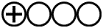  Very low |
| Power (personal value) | Obstetric practices | Cross-sectional (2) | 1 | Serious (-1) | None (0) | Serious (-1) | Serious (-1) | None (0) | 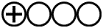  Very low |
| Benevolence (personal value) | Obstetric practices | Cross-sectional (2) | 1 | Serious (-1) | None (0) | Serious (-1) | Serious (-1) | None (0) | 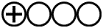  Very low |
| Self-direction (personal value) | Obstetric practices | Cross-sectional (2) | 1 | Serious (-1) | None (0) | Serious (-1) | Serious (-1) | None (0) | 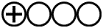  Very low |
| Stimulation (personal value) | Obstetric practices | Cross-sectional (2) | 1 | Serious (-1) | None (0) | Serious (-1) | Serious (-1) | None (0) | 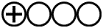  Very low |
| Achievement (personal value) | Obstetric practices | Cross-sectional (2) | 1 | Serious (-1) | None (0) | Serious (-1) | Serious (-1) | None (0) | 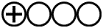  Very low |
| Tradition (personal value) | Obstetric practices | Cross-sectional (2) | 1 | Serious (-1) | None (0) | None (0) | Serious (-1) | None (0) | 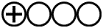  Very low |
| Hedonism (personal value) | Obstetric practices | Cross-sectional (2) | 1 | Serious (-1) | None (0) | Serious (-1) | None (0) | None (0) | 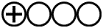  Very low |
| Universalism (personal value) | Obstetric practices | Cross-sectional (2) | 1 | Serious (-1) | None (0) | Serious (-1) | Serious (-1) | None (0) | 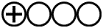  Very low |
| Conformity (personal value) | Obstetric practices | Cross-sectional (2) | 1 | Serious (-1) | None (0) | Serious (-1) | Serious (-1) | None (0) | 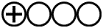  Very low |
|  |  |  |  |  |  |  |  |  |  |
| **Effect of the scope of practice on family physician performance**  Population: Family physicians  Exposures: Scope of practice  Outcomes: Family physician performance  Settings: Primary care and hospital; Urban, Semi urban, Rural; Clinics and hospitals | | | | | | | | | |
| Outcomes | Exposure | Study design | N of studies | Risk of bias | Inconsistency | Indirectness | Imprecision | Publication bias | Overall score |
| Quality of antibiotic prescribing | Public and private practice | Cohort | 1 | Serious (-1) | None (0) | None (0) | Serious (-1) | None (0) | 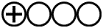  Very low |
| Quality of antibiotic prescribing | Hospital and primary care settings | Cohort | 1 | Serious (-1) | None (0) | None (0) | Serious (-1) | None (0) | 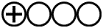  Very low |
| Quality of antibiotic prescribing | Emergency activity | Cohort | 1 | Serious (-1) | None (0) | None (0) | Serious (-1) | None (0) | 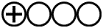  Very low |
| Providing IUD insertion | Performs Endometrial Biopsies | Cross-sectional (2) | 1 | Serious (-1) | None (0) | Serious (-1) | None (0) | None (0) | 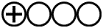  Very low |
| Providing Long-acting contraception insertion or removal | Performs Endometrial Biopsies | Cross-sectional (2) | 1 | Serious (-1) | None (0) | Serious (-1) | None (0) | None (0) | 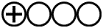  Very low |
| Providing IUD insertion | Performs Implant Insertions and Removals | Cross-sectional (2) | 1 | Serious (-1) | None (0) | Serious (-1) | None (0) | None (0) | 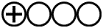  Very low |
| Providing Long-acting contraception insertion or removal | Performs IUD insertion | Cross-sectional (2) | 1 | Serious (-1) | None (0) | Serious (-1) | None (0) | None (0) | 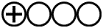  Very low |
| Providing IUD insertion | Performs Skin Procedures | Cross-sectional (2) | 1 | Serious (-1) | None (0) | Serious (-1) | None (0) | None (0) | 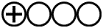  Very low |
| Providing Long-acting contraception insertion or removal | Performs Skin Procedures | Cross-sectional (2) | 1 | Serious (-1) | None (0) | Serious (-1) | None (0) | None (0) | 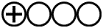  Very low |
| Providing IUD insertion | Provides prenatal care and deliveries | Cross-sectional (2) | 1 | Serious (-1) | None (0) | Serious (-1) | None (0) | None (0) | 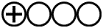  Very low |
| Providing Long-acting contraception insertion or removal | Provides prenatal care and deliveries | Cross-sectional (2) | 1 | Serious (-1) | None (0) | Serious (-1) | None (0) | None (0) | 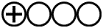  Very low |
| Providing IUD insertion | Provides prenatal care, no deliveries | Cross-sectional (2) | 1 | Serious (-1) | None (0) | Serious (-1) | None (0) | None (0) | 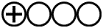  Very low |
| Providing Long-acting contraception insertion or removal | Provides prenatal care, no deliveries | Cross-sectional (2) | 1 | Serious (-1) | None (0) | Serious (-1) | None (0) | None (0) | 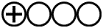  Very low |
| Managing patients with chronic conditions (chronic) | Focused practice scope | Cross-sectional (2) | 1 | Borderline (-0.5) | None (0) | None (0) | Serious (-1) | None (0) | 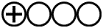  Very low |
| Providing patients with continuity of care and referrals (continuity) | Focused practice scope | Cross-sectional (2) | 1 | Borderline (-0.5) | None (0) | None (0) | Serious (-1) | None (0) | 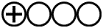  Very low |
| Providing patients with well care and health maintenance (well care) | Focused practice scope | Cross-sectional (2) | 1 | Borderline (-0.5) | None (0) | None (0) | Serious (-1) | None (0) | 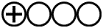  Very low |
| Managing patient records (records) | Focused practice scope | Cross-sectional (2) | 1 | Borderline (-0.5) | None (0) | None (0) | Serious (-1) | None (0) | 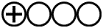  Very low |
| Managing patients with acute conditions and new presentations (acute) | Focused practice scope | Cross-sectional (2) | 1 | Borderline (-0.5) | None (0) | None (0) | Serious (-1) | None (0) | 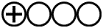  Very low |
| Managing patients with chronic conditions (chronic) | Holds Active Hospital appointment | Cross-sectional (2) | 1 | Borderline (-0.5) | None (0) | None (0) | Serious (-1) | None (0) | 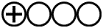  Very low |
| Providing patients with continuity of care and referrals (continuity) | Holds Active Hospital appointment | Cross-sectional (2) | 1 | Borderline (-0.5) | None (0) | None (0) | Serious (-1) | None (0) | 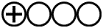  Very low |
| Providing patients with well care and health maintenance (well care) | Holds Active Hospital appointment | Cross-sectional (2) | 1 | Borderline (-0.5) | None (0) | None (0) | Serious (-1) | None (0) | 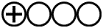  Very low |
| Managing patient records (records) | Holds Active Hospital appointment | Cross-sectional (2) | 1 | Borderline (-0.5) | None (0) | None (0) | Serious (-1) | None (0) | 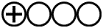  Very low |
| Managing patients with chronic conditions (acute) | Holds Active Hospital appointment | Cross-sectional (2) | 1 | Borderline (-0.5) | None (0) | None (0) | Serious (-1) | None (0) | 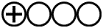  Very low |
| Managing patients with chronic conditions (chronic) | Episodic care practice/walk-in clinic | Cross-sectional (2) | 1 | Borderline (-0.5) | None (0) | None (0) | None (0) | None (0) | 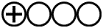  Very low |
| Providing patients with continuity of care and referrals (continuity) | Episodic care practice/walk-in clinic | Cross-sectional (2) | 1 | Borderline (-0.5) | None (0) | None (0) | Serious (-1) | None (0) | 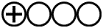  Very low |
| Providing patients with well care and health maintenance (well care) | Episodic care practice/walk-in clinic | Cross-sectional (2) | 1 | Borderline (-0.5) | None | None (0) | Serious (-1) | None (0) | 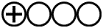  Very low |
| Managing patient records (records) | Episodic care practice/walk-in clinic | Cross-sectional (2) | 1 | Borderline (-0.5) | None | None (0) | Serious (-1) | None (0) | 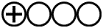  Very low |
| Managing patients with acute conditions and new presentations (acute) | Episodic care practice/walk-in clinic | Cross-sectional (2) | 1 | Borderline (-0.5) | None | None (0) | Serious (-1) | None (0) | 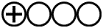  Very low |
| Offering maternal serum screening to all pregnant patients | Perform deliveries | Cross-sectional (2) | 1 | Borderline (-0.5) | None (0) | Serious (-1) | None (0) | None (0) | 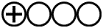  Very low |
| Offering maternal serum screening to some pregnant patients | Perform deliveries | Cross-sectional (2) | 1 | Borderline (-0.5) | None (0) | Serious (-1) | None | None (0) | 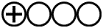  Very low |
| Time spent at work | Teaching | Cross-sectional (2) | 1 | Serious (-1 | None (0) | None (0) | Serious (-1) | None (0) | 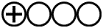  Very low |
| Number of patients seen per hour | Teaching | Cross-sectional (2) | 1 | Serious (-1) | None (0) | None (0) | Serious (-1) | None (0) | 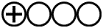  Very low |
| *The certainty was rated as following:   - Downgrading from +4 to +2 when the study design was observational studies (cohort or cross-sectional studies) - Downgrading Risk of bias, inconsistency, indirectness, imprecision and publication bias according their ratings none (score=0), borderline (-0.5), serious (score=-1), very serious (-2) - Upgrading (+1), when the association measure was strong and showed a dose-response relationship in the absence of plausible residual confounding bias.   **No score was upgraded because of the exploratory nature of statistical analyses performed in the studies identified.**  Score= 4 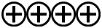 **High** = This research provides a very good indication of the likely effect. The likelihood that the effect will be substantially different** is low.  Score= 3 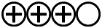 **Moderate** = This research provides a good indication of the likely effect. The likelihood that the effect will be substantially different** is moderate.  Score=2 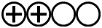 **Low** = This research provides some indication of the likely effect. However, the likelihood that it will be substantially different** is high.  Score= 1 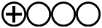 **Very low** = This research does not provide a reliable indication of the likely effect. The likelihood that the effect will be substantially different** is very high.  ** Substantially different = a large enough difference that it might affect a decision  *Schünemann HJ, Higgins JPT, Vist GE, Glasziou P, Akl EA, Skoetz N, Guyatt GH. Chapter 14: Completing ‘Summary of findings’ tables and grading the certainty of the evidence. In: Higgins JPT, Thomas J, Chandler J, Cumpston M, Li T, Page MJ, Welch VA (editors). Cochrane Handbook for Systematic Reviews of Interventions version 6.1 (updated September 2020). Cochrane, 2020. Available from*[*www.training.cochrane.org/handbook*](http://www.training.cochrane.org/handbook) | | | | | | | | | |
